# TAAR1 agonist ulotaront modulates striatal and hippocampal glutamate function in a state-dependent manner

**DOI:** 10.1038/s41386-023-01779-x

**Published:** 2023-12-19

**Authors:** Sung M. Yang, Ayan Ghoshal, Jeffrey M. Hubbard, Florian Gackière, Romain Teyssié, Stuart A. Neale, Seth C. Hopkins, Kenneth S. Koblan, Linda J. Bristow, Nina Dedic

**Affiliations:** 1 Sumitomo Pharma America, Inc., Marlborough, MA USA; 2 Neuroservices Alliance, Le Puy Sainte Réparade, France; 3 Neurexpert Limited, Newcastle, UK

**Keywords:** Cellular neuroscience, Preclinical research, Pharmacology

## Abstract

Aberrant dopaminergic and glutamatergic function, particularly within the striatum and hippocampus, has repeatedly been associated with the pathophysiology of schizophrenia. Supported by preclinical and recent clinical data, trace amine-associated receptor 1 (TAAR1) agonism has emerged as a potential new treatment approach for schizophrenia. While current evidence implicates TAAR1-mediated regulation of dopaminergic tone as the primary circuit mechanism, little is known about the effects of TAAR1 agonists on the glutamatergic system and excitation-inhibition balance. Here we assessed the impact of ulotaront (SEP-363856), a TAAR1 agonist in Phase III clinical development for schizophrenia, on glutamate function in the mouse striatum and hippocampus. Ulotaront reduced spontaneous glutamatergic synaptic transmission and neuronal firing in striatal and hippocampal brain slices, respectively. Interestingly, ulotaront potentiated electrically-evoked excitatory synaptic transmission in both brain regions, suggesting the ability to modulate glutamatergic signaling in a state-dependent manner. Similar striatal effects were also observed with the TAAR1 agonist, RO5166017. Furthermore, we show that ulotaront regulates excitation-inhibition balance in the striatum by specifically modulating glutamatergic, but not GABAergic, spontaneous synaptic events. These findings expand the mechanistic circuit hypothesis of ulotaront and TAAR1 agonists, which may be uniquely positioned to normalize both the excessive dopaminergic tone and regulate abnormal glutamatergic function associated with schizophrenia.

## Introduction

Schizophrenia is a severe, chronic, and often disabling psychiatric disorder that affects approximately 1% of the global population [1]. It is characterized by three symptom domains including positive symptoms (e.g., hallucinations and delusions), negative symptoms (e.g., social withdrawal, avolition and anhedonia) and cognitive impairment (e.g., dysfunction in attention, working memory and executive function) [2–5]. Despite advances in our understanding of its pathophysiology, schizophrenia remains one of the most challenging diseases to treat due to the diversity of clinical symptoms, the heterogeneity of clinical responses, the side effects of current treatments, and its association with high morbidity and mortality.

While the etiology and fundamental pathology of schizophrenia remain unclear, a large body of evidence suggests that alterations in several neurotransmitter systems are involved in the pathophysiological processes leading to the formation of positive, negative and cognitive symptoms [6, 7]. Among these, the dopaminergic and glutamatergic circuits have received the most attention, although the GABAergic, serotonergic, cholinergic, and opioid systems have also been implicated [8–11]. The prevailing mechanistic hypothesis of schizophrenia has centered on dysregulated dopaminergic modulation of striatal function. This is based on a large body of preclinical and clinical research including recent evidence from positron emission tomography imaging studies demonstrating increased presynaptic striatal dopamine synthesis and release capacity in schizophrenia patients compared to healthy controls [7, 9, 12]. Notably, several studies suggest that this hyperdopaminergic activity may be largely driven by aberrant glutamatergic neurotransmission and dysregulated excitatory–inhibitory (E/I) balance in brain regions upstream of midbrain dopaminergic nuclei, including the prefrontal cortex and hippocampus [7, 11, 12]. Dysregulated glutamate signaling and E/I imbalance have also been proposed to underly cognitive impairment and negative symptoms [7].

To date, all approved antipsychotic drugs for the treatment of schizophrenia rely on the blockade of postsynaptic dopamine (D2) receptors, although to varying degrees [13]. While these agents manage the positive symptoms, likely by blocking the downstream effects of hyperdopaminergia, they do not address the presynaptic dysregulation nor the upstream deficits in E/I balance associated with the disease. In addition, the existing treatments are largely ineffective for negative and cognitive symptoms, and are associated with significant side effects including extrapyramidal symptoms (e.g., parkinsonism, tardive dyskinesia and catalepsy) and metabolic dysfunction (e.g., weight gain, hyperglycemia and dyslipidemia) [14–17]. Thus, the urgency to develop new, non-D2 medications with improved efficacy and/or tolerability is apparent.

The trace amine-associated receptor 1 (TAAR1) has recently emerged as a promising therapeutic target for the treatment of schizophrenia and other neuropsychiatric disorders. TAAR1 is a G-protein-coupled receptor (GPCR) that is broadly expressed throughout the brain, although at low levels [18–20]. Preclinical studies utilizing both TAAR1 knockout and TAAR1-overexpressing mice, as well as small molecule agonists and antagonists, have revealed TAAR1-mediated modulation of monoaminergic and glutamatergic circuits including effects on neuronal firing and neurotransmission [21–24]. In particular, TAAR1 agonists have generated significant interest as potential treatments for schizophrenia due to their robust antipsychotic-like effects in rodent models and prominent regulation of dopaminergic tone [25–29]. Ulotaront (SEP-363856), which is currently in Phase III clinical development, was the first TAAR1 agonist to demonstrate efficacy in a randomized, double-blind, placebo-controlled Phase II clinical trial in patients with an acute exacerbation of schizophrenia [30]. Although the mechanism of action of ulotaront in the treatment of schizophrenia is not fully elucidated, preclinical studies suggest that activation of TAAR1 may normalize hyperdopaminergic activity by decreasing presynaptic dopamine synthesis capacity, release, and/or neuronal firing [31]. However, the effects of TAAR1 agonists, including ulotaront, on the glutamatergic system and E/I balance remain largely unexplored.

Here we show that ulotaront reduces spontaneous glutamatergic synaptic transmission in the dorsal striatum and neuronal firing in hippocampal CA1. Interestingly, ulotaront is able to potentiate electrically-evoked synaptic transmission in both brain regions, with the striatal effect being specific to a subpopulation of medium spiny neurons (MSNs). The ability of ulotaront to modulate glutamatergic activity in a state-dependent manner (i.e., selectively potentiate the evoked synaptic response while reducing the spontaneous activity) may facilitate an enhancement of the signal-to-noise ratio (SNR) in these brain regions. Ulotaront also regulates the E/I balance in the striatum by exclusively modulating glutamatergic, but not GABAergic, spontaneous synaptic transmission. The current results, together with prior data, suggest that ulotaront and TAAR1 agonists might regulate the complex interplay between the dopaminergic and glutamatergic systems to improve the reduced signal-to-noise ratio and presynaptic dopamine dysfunction in schizophrenia patients.

## Materials and methods

### Animals

Acute brain slices for electrophysiological recordings were obtained from 8- to 12-week-old Drd1a-tdTomato and 6- to 8-week-old C57BL/6J mice for striatal and hippocampal experiments, respectively. Animals used in the striatal experiments and hippocampal MEA recordings were treated according to the European guidelines 2010/63/UE and approved by the Animal Ethical Committee (Comité d’Ethique 71 pour l’Expérimentation Animale “Laurent Vinay” - CE71 from Neuroscience Institute of La Timone), with accreditation by the French Ministry of Education and Research (MESR). Animals for the remaining experiments were treated in accordance with the experimental conditions and procedures of the UK Animals (Scientific Procedures) Act 1986 and associated guidelines, and in compliance with the ARRIVE guidelines.

### Electrophysiological recordings

Whole-cell patch-clamp (voltage-clamp mode) recordings in the dorsal striatum were performed to investigate synaptic transmission. Extracellular recordings in the hippocampal CA1 region were performed to assess firing activity and synaptic transmission.

#### Striatum recordings

Acute coronal brain slices (300 μm thickness) containing the dorsal striatum were continually perfused with artificial Cerebro-Spinal Fluid (aCSF) and miniature postsynaptic currents were pharmacologically isolated for recording at 25 ± 1 °C. To record evoked excitatory postsynaptic currents (evEPSC), electrical stimulation was performed using a tungsten bipolar electrode placed in the deep cortical layer, close to the white matter (corpus callosum). The intensity of stimulation was set to elicit ~40% of the maximal evEPSCs amplitude. Each sweep of the protocol, delivered at a frequency of 0.05 Hz, integrated a dual stimulation (paired-pulse stimulation for paired-pulse ratio (PPR) evaluation; interstimulus interval of 50 ms) to elicit the evEPSC. Evoked-EPSCs were recorded at 30 ± 1 °C in the presence of 20 µM bicuculline to block GABA_A_ receptors.

#### Hippocampus recordings with MEA

During the recordings, coronal slices (300 μm thickness) containing the hippocampus were maintained at 32 °C. All data were recorded with a commercially available multi-electrode array (MEA) setup (Multichannel Systems GmbH), composed of 60 electrodes spaced by 100 µm. One line of electrodes was centered on the CA1 pyramidal cells. The threshold for detecting spikes was -4 times the standard deviation of the baseline noise. Only the electrodes displaying a steady firing rate greater than 0.5 Hz and stable over the 20-min baseline were validated (less than 15% of variation). For an electrode to be selected, it also needed to display at least 90% of firing rate inhibition in the presence of 1 µM tetrodotoxin (TTX).

#### Hippocampus recordings with glass pipette

Acute parasagittal brain slices (400 μm thickness) containing the hippocampus were maintained at 32−33 °C. Extracellular field potential recordings were made via a glass micropipette (resistance ~5 MΩ; filled with recording solution) positioned in the stratum pyramidale (for spontaneous neuronal firing) or stratum radiatum (for field excitatory post-synaptic potential; fEPSP) of the CA1.

Field EPSP responses were evoked by a bipolar stimulation electrode positioned in the stratum radiatum near the CA3-CA1 border. The stimulation electrode was used to deliver a pair of 0.02-ms pulses, separated by 40 ms, and applied every 10 s; the intensity of stimulation was adjusted to elicit approximately 60% of the maximal spike-free response.

More information on all experimental methods including brain slice preparation, recording conditions, solution and compound preparation, and data analysis are available in detail in the Supplementary Materials and Methods.

## Results

### Ulotaront decreases mEPSC frequency in putative D2 MSNs

**Fig. 1:**
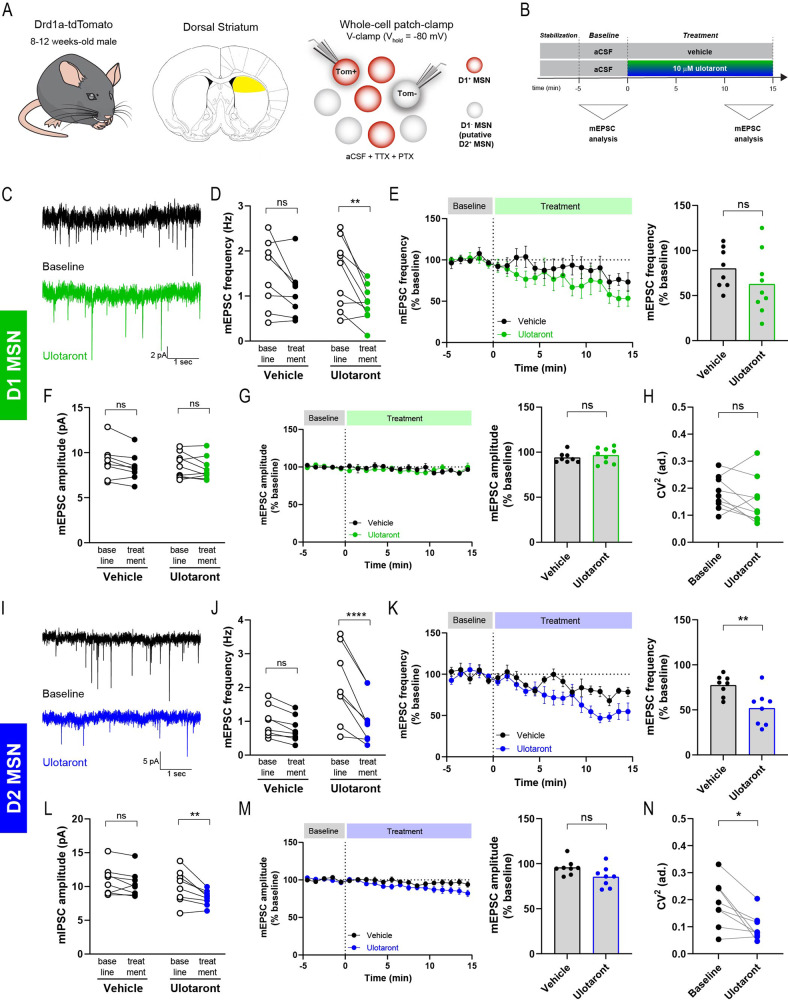
Ulotaront decreases the frequency of mEPSC in striatal MSNs. **A** Schematics of the mouse line (left), brain area (middle) and recording strategy (right). mEPSCs were recorded in D1-expressing (tdTom + ) and D1-non-expressing (tdTom-) MSNs using whole-cell V-clamp configuration and with TTX and PTX added into the aCSF. **B** Experimental time course. Frequency and amplitude of mEPSCs were analyzed during pre-drug baseline condition and for the last 5 min of treatment with 10 μM ulotaront or vehicle. **C**−**H** mEPSC in D1-expressing MSNs. **C** Representative traces of mEPSC activity before (top) and during ulotaront application (bottom). Average frequency (**D**) and amplitude (**F**) of mEPSC during baseline condition and upon bath application of 10 μM ulotaront or vehicle. Effect of treatment with vehicle control vs ulotaront on frequency (**E**) and amplitude (**G**) of mEPSC. Data were normalized to baseline and presented as average time course (left) as well as summary plot (right). **H** Coefficient of variation analysis for the treatment with ulotaront. **I**−**N** Analysis on D1-non-expressing MSNs (putative D2-expressing), corresponding to (**C**−**H**). Data are mean ± s.e.m. Each dot represents a cell (*n* = 8−9), except in the average time course (**E**, **G**, **K** and **M**, left). Statistics are described in Table S1. ns, not significant; *p < 0.05; **p < 0.01.

Medium spiny neurons (MSNs) constitute the largest neuronal population and are the main projection neurons of the striatum. They are characterized by their rich dendritic spines which receive cortical and thalamic glutamatergic inputs. A distinct feature of MSNs is the segregation into dopamine D_1_ and D_2_ receptor-expressing subpopulations, which constitute the direct and indirect pathway MSNs, respectively [32]. To investigate the effects of ulotaront on synaptic transmission in MSNs of the dorsal striatum, we performed experiments on acute brain slices from adult Drd1a-tdTomato mice which express the fluorescence protein tdTomato under the control of the dopamine 1 receptor (D1) promotor (Fig. 1A). MSNs were identified based on their electrophysiological properties (see Supplementary Materials and Methods; briefly, voltage “sag”, hyperpolarized resting membrane potential and regular firing), and classified depending on the presence or absence of tdTomato expression: D1-expressing MSNs (tdTomato^+^; D1 MSN) and D1-non-expressing MSNs (tdTomato^**-**^; putative D2 MSN or, shortly, D2 MSN).

Miniature excitatory postsynaptic currents (mEPSC) were recorded in whole-cell patch-clamp configuration (V_h_ = -80 mV) and pharmacologically isolated by adding 500 nM TTX and 50 μM picrotoxin (PTX) to the aCSF (Fig. 1A). Furthermore, the mEPSC events were abolished upon bath application of 30 μM D-AP5 (D-2-amino-5-phosphonopentanoate) and 10 μM NBQX (2,3-dioxo-6-nitro-1,2,3,4-tetrahydrobenzo[f]quinoxaline-7-sulfonamide), antagonists of NMDA and non-NMDA glutamate receptors, respectively, indicating that the recorded mEPSCs were mediated by glutamatergic excitatory synapses (Supplementary Fig. 1A, B; *n* = 3 cells).

To assess the effect of ulotaront on mEPSCs, the baseline synaptic activity was recorded for 5 min followed by bath application of 10 μM ulotaront or vehicle control (0.15% DMSO) (Fig. 1B). In D1 MSNs, mEPSC frequency showed a decrease upon ulotaront application compared to the baseline condition (Fig. 1C, D; Supplementary Fig. 1C; baseline, 1.50 ± 0.26 Hz vs ulotaront, 0.84 ± 0.13 Hz). However, the decrease induced by ulotaront was not significantly different from the effect in the vehicle group (Fig. 1D; 2-way ANOVA: time factor, *p* = 0.0008 and “time x compound” interaction, *p* = 0.273). In line with this analysis, the normalized mEPSC frequency (relative to baseline) upon application of ulotaront was not significantly different from the vehicle control (Fig. 1E; vehicle, 80.1 ± 8.0% vs ulotaront, 62.7 ± 11.3%).

In contrast, when we analyzed mEPSC frequency in putative D2 MSNs, ulotaront elicited a significant decrease compared to the baseline condition (Fig. 1I, J; Supplementary Fig. 1D; baseline, 2.06 ± 0.39 Hz vs ulotaront, 1.05 ± 0.25 Hz) as well as to the vehicle control (Fig. 1J; 2-way ANOVA: time factor, *p* < 0.0001 and “time x compound” interaction, *p* = 0.0015; Fig. 1K; vehicle, 77.3 ± 4.0% vs ulotaront, 51.9 ± 6.8%).

Altogether, these results indicate that ulotaront significantly reduces the frequency of mEPSC in putative D2 MSNs, with a similar, statistically non-significant, trend observed in D1 MSNs.

### Ulotaront does not significantly affect mEPSC amplitude in MSNs

After evaluating the effect of ulotaront on the frequency of mEPSCs generated in MSNs of the dorsal striatum, we analyzed the amplitude of those events and their modulation by ulotaront. In D1 MSNs, 10 μM ulotaront did not alter the amplitude of mEPSCs when compared to baseline (Fig. 1F, Supplementary Fig. 1E; baseline, 8.56 ± 0.46 pA vs ulotaront, 8.23 ± 0.42 pA) or vehicle control (Fig. 1G; vehicle, 93.9 ± 2.0% vs ulotaront, 96.6 ± 2.8%). In line with the lack of effect of ulotaront on mEPSC amplitude of D1 MSNs, the coefficient of variation (CV^2^; Fig. 1H, baseline 0.18 ± 0.02 vs ulotaront, 0.15 ± 0.03) and the variance-to-mean ratio (VMR; Supplementary Fig. 1H) did not change upon bath application of ulotaront, in similar fashion to the result of the vehicle group (Supplementary Fig. 1G, H; CV^2^: baseline, 0.20 ± 0.03 vs vehicle, 0.16 ± 0.03).

We then performed a similar analysis on mEPSC amplitude of D2 MSNs. In this neuronal subpopulation, application of 10 μM ulotaront induced a decrease in mEPSC amplitude compared to the pre-drug baseline condition (Fig. 1L, Supplementary Fig. 1F; baseline 10.03 ± 0.87 vs ulotaront, 8.32 ± 0.40). However, the decrease induced by ulotaront was not significantly different from the effect in the vehicle group (Fig. 1L; 2-way ANOVA: time factor, *p* = 0.133; compound factor, *p* = 0.002 and “time x compound” interaction, *p* = 0.064). Furthermore, there was no significant difference in normalized mEPSC amplitude between vehicle and ulotaront treatments (Fig. 1M; vehicle, 95.9 ± 3.1% vs ulotaront, 85.3 ± 4.0%).

In line with the effect on mEPSC frequency (Fig. 1J, K), the coefficient of variation and the variance-to-mean ratio in D2 MSNs were altered by bath application of ulotaront (Fig. 1N; CV^2^: baseline 0.19 ± 0.03 vs ulotaront, 0.10 ± 0.02; Supplementary Fig. 1J), in contrast to the absence of effect on CV^2^ generated by the vehicle (Supplementary Fig. 1I; CV^2^: baseline, 0.21 ± 0.04 vs vehicle, 0.19 ± 0.04).

Altogether, these results suggest that the effect of ulotaront on mEPSCs of D2 MSNs relies primarily on presynaptic mechanisms that alter their frequency.

### Ulotaront modulates the E/I balance in putative D2 MSNs

Given that ulotaront exhibited significant effects on excitatory synaptic transmission in D2 MSNs, we then assessed whether it is also able to modify the inhibitory synaptic activity and further alter the excitation-to-inhibition balance (E/I) in the mouse dorsal striatum.

**Fig. 2:**
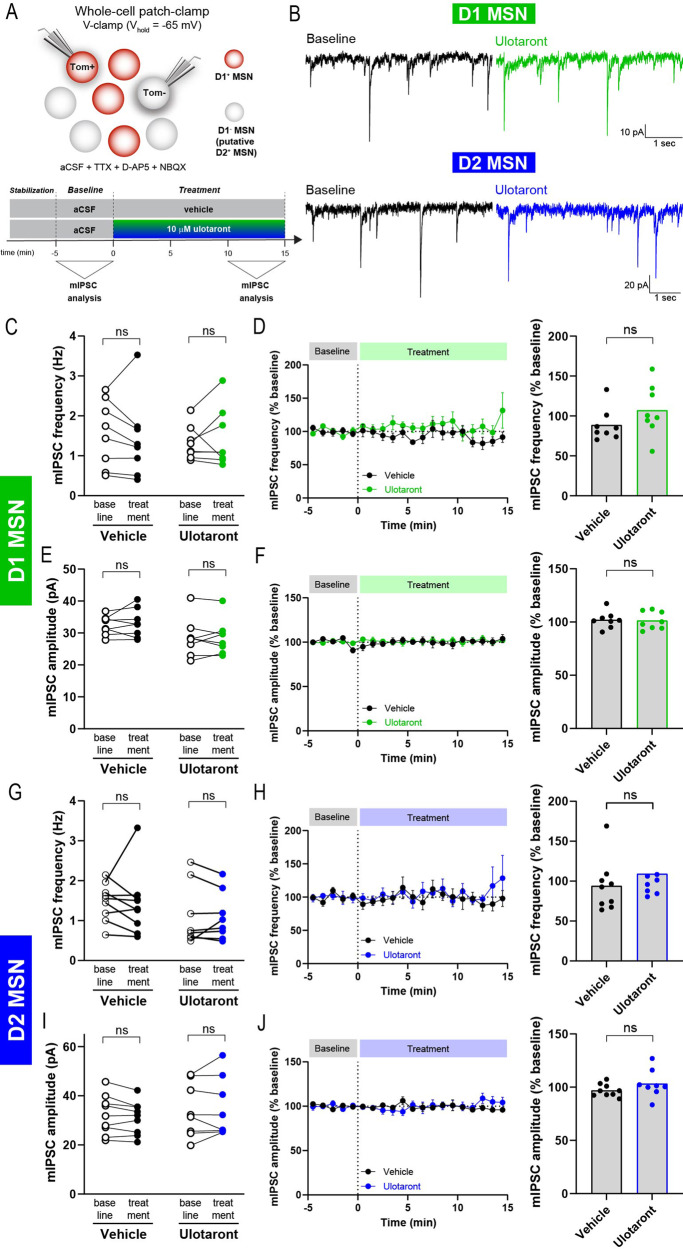
Ulotaront does not affect the mIPSC in striatal MSNs. **A** Schematics of recording strategy (top) and experimental time course (bottom). mIPSCs were recorded in D1-expressing (tdTom + ) and D1-non-expressing (tdTom-) MSNs using whole-cell V-clamp configuration and with TTX, D-AP5 and NBQX added into the aCSF. Frequency and amplitude of mIPSC were analyzed during pre-drug baseline condition and for the last 5 min of treatment with 10 μM ulotaront or vehicle. **B** Representative traces of mIPSC activity before (left) and during ulotaront application (right), for both D1-expressing (top) and D1-non-expressing (bottom) MSNs. **C**−**F** mIPSC in D1-expressing MSNs. Average frequency (**C**) and amplitude (**E**) of mIPSC during baseline and upon bath application of 10 μM ulotaront or vehicle. Effect of treatment with vehicle vs ulotaront on frequency (**D**) and amplitude (**F**) of mIPSC. Data were normalized to baseline and presented as time course (left) as well as summary plot (right; each dot represents a cell, as in (**C**, **E**)). **G**−**J** Analysis on D1-non-expressing (putative D2-expressing) MSNs, corresponding to (**C**−**F**). Data are mean ± s.e.m; *n* = 8 -9 slices. Statistics are described in Table S1. ns, not significant.

Using the same experimental preparation as for the mEPSC analysis, recordings in whole-cell voltage-clamp configuration (V_h_ = −65 mV) were performed to measure miniature inhibitory postsynaptic current (mIPSC), isolated by adding 500 nM TTX, 30 μM D-AP5 and 10 μM NBQX to the aCSF (Fig. 2A). These synaptic events were abolished upon bath application of 50 μM PTX, indicating that the recorded mIPSC were mediated by GABAergic synapses (Supplementary Fig. 2A,B; *n* = 3 cells).

We measured the mIPSC events in D1 and putative D2 MSNs (Fig. 2B) and analyzed the effect of bath application of 10 μM ulotaront compared to vehicle control (0.15% DMSO). In D1 MSNs, there was no change in frequency (Fig. 2C, D; Supplementary Fig. 2C) or amplitude (Fig. 2E,F; Supplementary Fig. 2E) of mIPSCs upon application of ulotaront compared to baseline (frequency: baseline, 1.32 ± 0.15 Hz vs ulotaront, 1.44 ± 0.26 Hz; amplitude: baseline, 28.5 ± 2.1 pA vs ulotaront, 28.7 ± 1.9 pA) or vehicle (frequency: vehicle, 88.6 ± 7.2% vs ulotaront, 107 ± 11%; amplitude: 101.9 ± 2.8% vs ulotaront, 101.3 ± 3.0%).

Similar to the observations in D1 MSNs, mIPSCs in putative D2 MSNs were not affected by bath application of ulotaront, both at the level of frequency (Fig. 2G, H; Supplementary Fig. 2D) and their amplitude (Fig. 2I, J; Supplementary Fig. 2F). The absence of effect was corroborated when compared to the pre-drug baseline condition (frequency: baseline, 1.11 ± 0.27 Hz vs ulotaront, 1.10 ± 0.21 Hz; amplitude: baseline, 34.1 ± 3.9 pA vs ulotaront, 35.0 ± 4.3 pA) as well as to the vehicle control (frequency: vehicle, 94 ± 11% vs ulotaront, 109 ± 15%; amplitude: 97.0 ± 2.0% vs ulotaront, 103.3 ± 4.6%). In line with these results, CV^2^ and VMR were not altered upon treatment with ulotaront (Supplementary Fig. 2G, H).

Taken together, these data indicate that bath application of ulotaront does not induce significant changes in mIPSC activity of MSNs (D1 and D2). The fact that ulotaront selectively altered spontaneous glutamatergic synaptic transmission (i.e., mEPSC frequency; Fig. 1) suggests that it modulates striatal E/I balance, in particular, in D2 MSNs.

### Ulotaront modulates evoked EPSC amplitude in putative D2 MSNs

Since bath application of ulotaront reduced the spontaneous excitatory synaptic transmission in the dorsal striatum, we hypothesized that ulotaront would also modulate the excitatory synaptic response elicited upon stimulation of the afferent pathway (i.e., cortico-striatal projections).

**Fig. 3:**
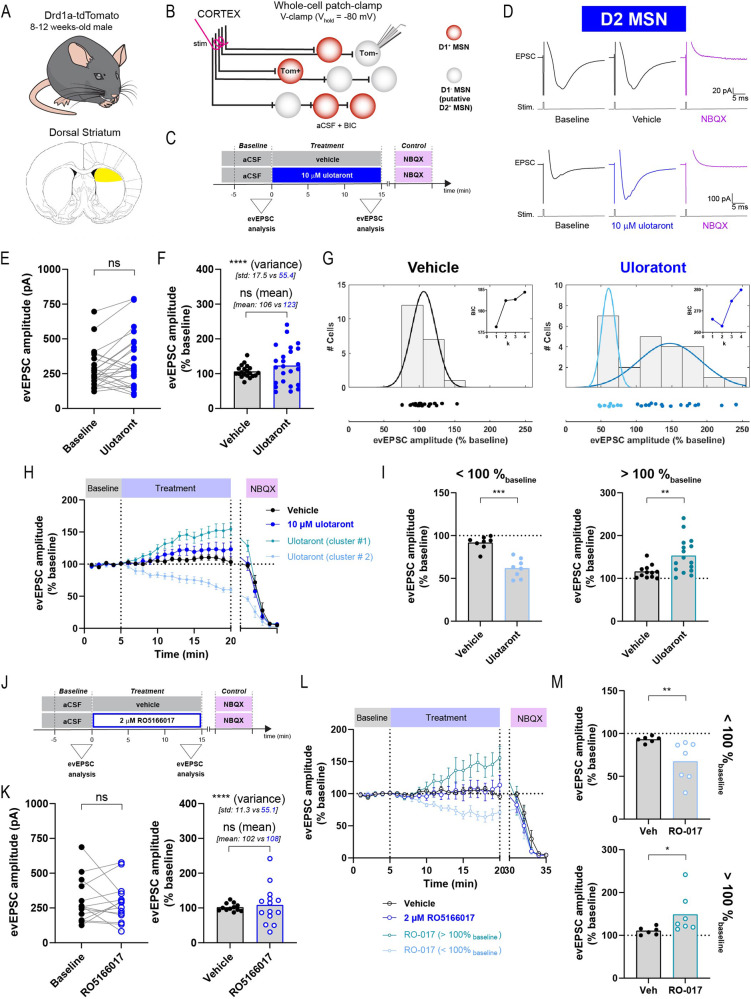
Ulotaront and selective TAAR1 agonist RO5166017 bidirectionally modulate the amplitude of evoked EPSC in putative D2 MSNs. Schematic of the mouse line (**A**, top), brain area (**A**, bottom) and recording strategy (**B**). Evoked EPSCs were elicited by stimulation of the deep cortical layer and recorded in D1-non-expressing (tdTom-) MSNs using whole-cell V-clamp configuration, with bicuculline (BIC) added into the aCSF. **C**−**H** Effect of ulotaront on evEPSC in putative D2-expressing MSNs. **C** Experimental time course. Evoked EPSC amplitude was analyzed during pre-drug baseline condition and for the last 3 min of treatment with 10 μM ulotaront or vehicle. **D** Representative average traces of evEPSCs before and during vehicle (top) and ulotaront application (bottom), as well as upon perfusion with of 10 μM NBQX. **E** Amplitude during baseline condition and upon bath application of ulotaront. **F** Comparison of the normalized-to-baseline evEPSC amplitude for vehicle- vs ulotaront-treated groups, at the level of mean and variance (average mean and standard deviation are presented in parentheses). **G** Individual data (bottom) and count histogram (top) of the normalized amplitude for vehicle (left) and ulotaront (right) groups. The histogram is overlaid by the optimal Gaussian mixture model (number of components *k* = 1 and *k* = 2 for vehicle and ulotaront groups, respectively; see Table S1) and the individual values were color-coded based on a cluster analysis run on the distribution model (see Supplementary Materials and Methods). *Inset*, plot of the Bayes information criterion (BIC) generated for the Gaussian mixture models with k components. **H** Average time course of the normalized evEPSC amplitude. The ulotaront data is presented as a single group, and also split into the two clusters found in *G*. **I** Analysis of vehicle vs ulotaront groups after the cells were clustered based on the direction of the change relative to baseline; decrease (left, < 100%_baseline_) and increase (right, > 100%_baseline_). **J**−**M** Effect of 2 μM RO5166017 (RO-017) on evEPSC, corresponding to (**C**), (**E**), (**F**), (**H**) and (**I**). In (**L**) and (**M**), cells were grouped based on the direction of the effect; decrease (**M** top, <100%_baseline_) and increase (**M** bottom, >100%_baseline_) relative to baseline. Data are mean ± s.e.m. Each dot represents a cell (*n* = 7−24), except in the average time course (**H**, **L**). Statistics are described in Table S1. ns, not significant; *p < 0.05; **p < 0.01; ***p < 0.001; ****p < 0.0001.

To test this hypothesis, acute brain slices containing the dorsal striatum were obtained from Drd1a-tdTomato mice as in the previous experiments. EPSCs were elicited in D1 and putative D2 MSNs via electrical stimulation of the afferent pathway coming from the cortex using an electrode placed in the deep cortical layer, close to the corpus callosum (Fig. 3A, B). The evoked EPSC (evEPSC) were evaluated in whole-cell voltage-clamp mode (V_h_ = −80 mV) and pharmacologically isolated by adding 20 μM bicuculline in the aCSF to block GABA_A_ receptors. Ulotaront was tested at 10 μM for 15 min, with 5 min of pre-drug baseline recording (Fig. 3C). The evEPSCs were abolished upon bath application of 10 μM NBQX, indicating that the response was mediated by glutamatergic synapses (Fig. 3D).

In putative D2 MSNs, ulotaront elicited both increases and decreases in evEPSC amplitude compared to baseline, on a cell-by-cell basis, but with an overall lack of significant effect at the population level (Fig. 3E; baseline, 283 ± 28 pA vs ulotaront, 335 ± 40 pA). In line with this result, when the evEPSC amplitude was normalized to baseline and compared to vehicle control, ulotaront induced a significant expansion in the data dispersion (standard deviation: vehicle, 17.5% vs ulotaront, 55.4%) but not in its mean value (Fig. 3F; vehicle, 106.2 ± 3.9% vs ulotaront, 122.8 ± 11.3%). In fact, by applying Gaussian mixture models combined with minimization of the Bayes information criterion, we found that the optimal number of components (or subpopulations) was one for the vehicle groups, whereas for the ulotaront group it was two. Furthermore, the optimal Gaussian mixture model for the ulotaront group divided the data in two distinct subpopulations: cells showing an increase (>100%_baseline_) or a decrease (<100%_baseline_) in evEPSC amplitude relative to baseline (Fig. 3G, H). Importantly, when each subpopulation was compared to its corresponding vehicle subgroup (>100%_baseline_ or <100%_baseline_), the analysis indicated a cell-type-specific effect of ulotaront on evEPSC amplitude in putative D2 MSNs (Fig. 3I): a significant increase in one subpopulation (vehicle, 115.8 ± 4.5% vs ulotaront, 153.2 ± 10.2%, 16 out of 24 neurons), as well as a significant decrease in a different subset of D2 MSNs (vehicle, 91.7 ± 2.7% vs ulotaront, 61.9 ± 3.9%, 8 out of 24 cells).

In D1 MSNs, ulotaront also induced no change in evEPSC amplitude compared to baseline (Supplementary Fig. 3A−C; baseline, 269 ± 42 pA vs ulotaront, 345 ± 77 pA). Similar to the observation in putative D2 MSNs, ulotaront increased the variance of the normalized-to-baseline amplitude (standard deviation: vehicle, 19.5% vs ulotaront, 60.5%) without altering the population mean (vehicle, 104.7 ± 5.9% vs ulotaront, 129.7 ± 19.1%) in D1 MSNs (Supplementary Fig. 3D, E). However, after analyzing separately the cells showing an increase (vehicle, 115.7 ± 5.2% vs ulotaront, 154 ± 21%, 7 out of 10 neurons) or a decrease (vehicle, 85.5 ± 5.2% vs ulotaront, 72.8 ± 1.8%, 3 out of 10 neurons) relative to baseline, ulotaront showed only a non-significant trend towards a cell-specific effect compared to vehicle in evEPSC amplitude of D1 MSNs (Supplementary Fig. 3F).

Analysis of the kinetic properties of the evEPSC showed that ulotaront did not induce significant changes in the rise or decay times in both D1 and D2 MSNs (Supplementary Fig. 4A−D). In line with the effect on evEPSC amplitude, bath application of ulotaront did not alter the paired-pulse ratio (PPR) in D1 MSNs, but induced an increase in the dispersion of normalized-to-baseline PPR in putative D2 MSNs (Supplementary Fig. 4E−H).

Evaluation of evoked synaptic responses showed that ulotaront bidirectionally modulates cortico-striatal evEPSC amplitude in MSNs of the dorsal striatum. Consistent with its effects on spontaneous miniature excitatory synaptic transmission (mEPSC), more prominent modulation of evoked responses was observed in putative D2 MSNs.

### TAAR1 agonist RO5166017 modulates evoked EPSC amplitude in putative D2 MSNs

In addition to TAAR1 agonism, ulotaront also exhibits agonist activity at the serotonin 5-HT_1A_ receptor [28, 29]. We therefore investigated the selective TAAR1 agonist, RO5166017, to determine whether this agent produced effects on excitatory glutamatergic synaptic transmission similar to those seen with ulotaront. Given the more pronounced effect of ulotaront on putative D2 MSNs, we focused our analysis on this neuronal subpopulation.

Briefly, we used the Drd1a-tdTomato mice to identify putative D2 MSNs for patch-clamp recording of evEPSC triggered by electrical stimulation of the cortical afferent pathway (Fig. 3A, B). RO5166017 was tested at 2 μM for 15 min, with 5 min of pre-drug baseline recording (Fig. 3J). Here again, the glutamatergic origin of the response was validated by bath application of 10 μM NBQX (Fig. 3L). Similar to the effect of ulotaront, bath application of RO5166017 induced both increases and decreases in evEPSC amplitude, that were larger in size than the vehicle control group and were reflected in a significant expansion of the data distribution (standard deviation: vehicle, 11.3% vs RO5166017, 55.1%; Fig. 3K). RO5166017 did not affect the population average response, both when compared to baseline (baseline, 299 ± 43 pA vs RO5166017, 286 ± 39 pA) or to the vehicle group (vehicle, 101.8 ± 3.3% vs RO5166017, 108.1 ± 14.7%,). Similar to the analysis performed for ulotaront, we then divided the cells based on the effect direction: increase ( > 100%_baseline_) or decrease ( < 100%_baseline_) compared to baseline, for both the vehicle and the RO5166017 groups (Fig. 3L). This evaluation showed that RO5166017 induced changes in evEPSC amplitude that were significantly larger than vehicle for both the increasing (vehicle, 110.5 ± 3.7% vs RO5166017, 148.8 ± 17.6%, 7 out of 14 neurons) and the decreasing (vehicle, 93.1 ± 1.5% vs RO5166017, 67.5 ± 8.8%, 7 out of 14 neurons) subpopulation (Fig. 3M). Furthermore, the similarity between ulotaront and TAAR1 selective agonist RO5166017 was not only limited to the cell-specific bidirectionality of the effect, but also reflected in the size of the effect ( > 100%_baseline_: ulotaront, +53.2 ± 10.2% vs RO5166017, +48.8 ± 17.6%; <100%_baseline_: ulotaront, −38.1 ± 3.9% vs RO5166017, −32.5 ± 8.8%) and proportion of cells in each cluster (>100%_baseline_: ulotaront, 66.7% [16/24 cells] vs RO5166017, 50% [7/14 cells]; <100%_baseline_: ulotaront, 33.3% [8/24 cells] vs RO5166017, 50% [7/14 cells]).

Collectively, these results demonstrate that the TAAR1 agonists ulotaront and RO5166017 induce similar effects on evoked excitatory synaptic transmission in the dorsal stratum, characterized by the presence of opposite modulation of evEPSC responses in two distinct subpopulations of D2 MSNs.

### Ulotaront enhances evoked fEPSP in hippocampal CA1

**Fig. 4:**
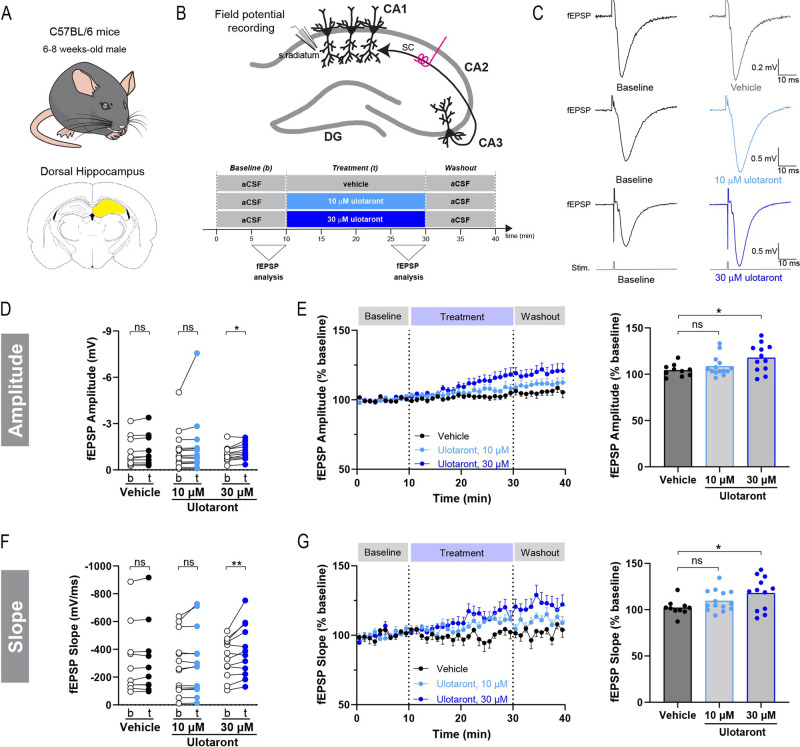
Ulotaront enhances excitatory synaptic transmission in hippocampal CA1. Schematics of animal model (**A**, top), brain area (**A**, bottom), recording strategy (**B**, top) and experimental time course (**B**, bottom). Field EPSP were measured in the stratum radiatum of the CA1 by means of extracellular field potential recordings upon stimulation of the Schaffer Collateral pathway (SC). Peak amplitude and initial slope of fEPSP were analyzed during pre-drug baseline condition and for the last 5 min of treatment with ulotaront (10 μM and 30 μM) or vehicle. **C** Representative average traces of fEPSP responses before (left) and during compound application (right). **D**, **E** Peak amplitude of fEPSP in CA1. **D** fEPSP amplitude during baseline condition (b) and upon bath application (t) of ulotaront (10 μM and 30 μM) or vehicle. **E** Effect of treatment with vehicle control vs ulotaront on fEPSP amplitude. Data were normalized to baseline and presented as average time course (left) as well as summary plot (right; each dot represents a brain slice, as in (**D**)). **F**, **G** Analysis on fEPSP slope, corresponding to (**D**, **E**). Data are mean ± s.e.m; *n* = 10 -14 slices. For statistics, see Table S1. ns, not significant; *p < 0.05.

In addition to the striatum, another brain area associated with the pathophysiology of schizophrenia is the hippocampus. Thus, we set out to investigate whether ulotaront also modulates hippocampal excitatory synaptic transmission. Acute brain slices containing the dorsal hippocampus were obtained from 6 to 7-week-old C57BL/6J mice (Fig. 4A). Field excitatory postsynaptic potentials (fEPSP) were recorded using a glass micropipette positioned in the stratum radiatum of the CA1 and upon stimulation of the Schaffer collateral pathway (Fig. 4B). To assess the effect of ulotaront on fEPSP responses, the baseline synaptic activity was recorded for 10 min followed by bath application of ulotaront or vehicle (aCSF) (Fig. 4B, C).

Bath application of ulotaront induced a concentration-dependent increase in both the peak amplitude and initial slope of the fEPSP with significant effects seen at 30 μM (Fig. 4D, amplitude: baseline, −1.05 ± 0.14 mV vs 30 μM ulotaront, −1.19 ± 0.15 mV; Fig. 4F, slope: baseline, −339 ± 40 mV/ms vs 30 μM ulotaront, −394 ± 54 mV/ms). The effect on fEPSP initial slope (associated with the excitatory input) was more pronounced, as reflected by the two-way ANOVA (time factor, *p* = 0.0026 and “time x compound” interaction, *p* = 0.048). When the data was normalized to baseline and then compared to the vehicle control group, the enhancement of the fEPSP response elicited upon application of ulotaront was corroborated for both the peak amplitude (Fig. 4E; vehicle, 104.2 ± 2.1% vs 10 μM ulotaront, 108.5 ± 2.9% vs 30 μM ulotaront, 117.6 ± 4.3%) and the initial slope (Fig. 4G; vehicle, 102.1 ± 2.7% vs 10 μM ulotaront, 109.5 ± 3.0% vs 30 μM ulotaront, 117.8 ± 5.2%). In addition, ulotaront did not alter the PPR of hippocampal fEPSP responses (Supplementary Fig. 5), suggesting that postsynaptic mechanisms might be involved.

Taken together, these data indicate that ulotaront enhances excitatory synaptic transmission in response to stimulation of the afferent pathway in hippocampal CA1.

### Ulotaront reduces spontaneous activity in hippocampal CA1

**Fig. 5:**
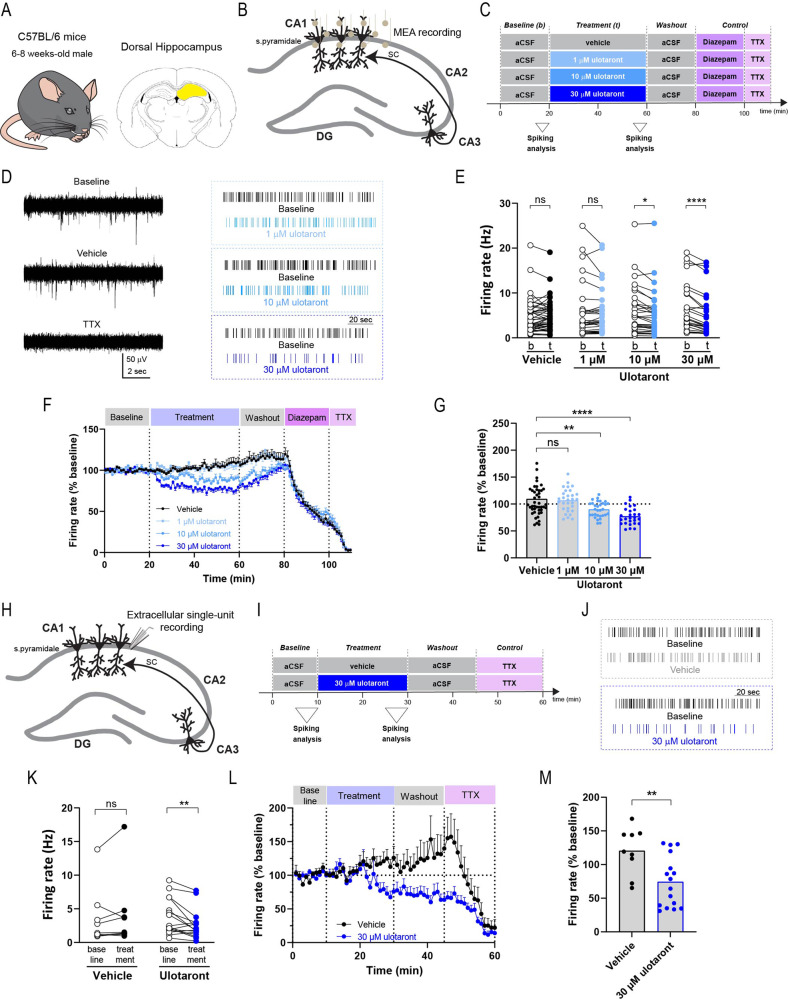
Ulotaront reduces spontaneous firing in hippocampal CA1. **A**−**G** Spontaneous firing in the CA1 recorded by multi-electrode array (MEA). Schematic of the mouse line (**A**, left), brain area (**A**, right), recording strategy (**B**) and experimental time course (**C**). Spontaneous neuronal firing activity was extracellularly recorded in the dorsal CA1 by means of a MEA. Firing rate was analyzed during pre-drug baseline condition and for the last 5 min of treatment with ulotaront (1 μM, 10 μM and 30 μM) or vehicle. **D** *Left*, Representative traces of spontaneous firing before (top) and during (middle) vehicle application, and upon TTX (bottom). *Right*, representative raster plots displaying the firing activity for the ulotaront groups (each line represents a spike). **E** Firing rate during baseline (b) and upon bath application (t) of ulotaront (1 μM, 10 μM, 30 μM) or vehicle. **F**, **G** Effect of treatment with vehicle control vs ulotaront on firing rate. Data were normalized to baseline and presented as average time course (**F**) as well as summary plot (**G**; each dot represents an electrode, as in (**E**)). **H**−**L** Spontaneous firing in the CA1 recorded by micropipette. **H**, **I** Recording strategy (**H**) and experimental time course (**I**). Extracellular single-unit recordings were acquired by means of a micropipette positioned in the stratum pyramidale of the CA1. Firing rate was analyzed during pre-drug baseline condition and for the last 5 min of treatment with 30 μM ulotaront or vehicle. **J** Representative raster plots displaying the spontaneous firing for the vehicle (top) and ulotaront (bottom) groups. **K**−**M** Analysis on firing rate recorded by micropipette, corresponding to (**E**−**G**). In (**K**) and (**M**), each dot represents a unit. Data are mean ± s.e.m; *n* = 27−45 electrodes, 9−16 cells. Statistics are described in Table S1. ns, not significant; **p < 0.01; ****p < 0.0001.

Since TAAR1 agonists have been shown to modulate the intrinsic excitability of different neuronal populations, including dopaminergic neurons in the ventral tegmental area (VTA) and serotonergic neurons in the dorsal raphe nucleus (DRN), we assessed the effect of ulotaront on spontaneous firing of hippocampal neurons using multielectrode array (MEA) placed on the CA1 (Fig. 5A, B). Ulotaront was tested at three different concentrations and compared to vehicle (Fig. 5C).

The spontaneous firing rate in normal aCSF was stable (Fig. 5D) and was decreased upon bath application of ulotaront in a concentration-dependent manner (Fig. 5E; two-way ANOVA, “time x compound” interaction, *p* = 0.0003), with significant effects observed at 10 μM and 30 μM compared to the pre-drug baseline (baseline, 5.96 ± 0.97 Hz vs 10 μM ulotaront, 5.29 ± 0.92 Hz; baseline, 6.68 ± 1.04 Hz vs 30 μM ulotaront, 5.46 ± 0.99 Hz). Ulotaront also decreased the rate of TTX-sensitive action potentials in a concentration-dependent manner compared to the vehicle control group (Fig. 5F, G; vehicle, 109.3 ± 5.7% vs 1 μM ulotaront, 106.6 ± 3.9% vs 10 μM ulotaront, 89.8 ± 2.6% vs 30 μM ulotaront, 77.0 ± 3.1%).

To confirm the effect of ulotaront on spontaneous firing at a single cell level, we performed extracellular single-unit recordings in the striatum pyramidale of CA1 (Fig. 5H) at the highest test concentration (30 μM; Fig. 5I). Similar to the MEA recordings, spontaneous firing rate measured by extracellular micropipette showed a decrease upon bath application of 30 μM ulotaront compared to baseline (Fig. 5J, K; baseline, 3.75 ± 0.67 Hz vs ulotaront, 2.58 ± 0.54 Hz), while application of vehicle did not affect the firing rate (baseline, 3.47 ± 1.4 Hz vs vehicle, 3.99 ± 1.7 Hz). Moreover, the comparison of the normalized rate of TTX-sensitive action potentials between the vehicle and the ulotaront treatment confirmed the reduction in spontaneous firing induced by ulotaront (Fig. 5L, M; vehicle, 120.3 ± 11.6% vs ulotaront, 74.4 ± 9.5%).

In summary, these results demonstrate that ulotaront reduces the spontaneous spiking activity in the hippocampal CA1.

## Discussion

TAAR1 is emerging as a potential new therapeutic target for schizophrenia and several other neuropsychiatric disorders. Current preclinical evidence suggests that the antipsychotic-like effects of TAAR1 agonists are linked to TAAR1-mediated regulation of dopaminergic tone, including reduction of presynaptic hyperdopaminergic activity [12, 32]. However, TAAR1 functional regulation of glutamate systems in schizophrenia-relevant brain areas such as striatum and hippocampus has been largely unexplored. In this study, we used acute slice electrophysiology to demonstrate that ulotaront, a TAAR1 agonist with 5-HT_1A_ agonist activity, reduces the spontaneous glutamatergic but not GABAergic synaptic events in D2 MSNs of the dorsal striatum. Ulotaront also exhibited a cell type-dependent, bidirectional effect on the amplitude of the glutamatergic synaptic response elicited in D2 MSNs by stimulation of the cortical afferent pathway. Notably, the same effect was observed with the selective TAAR1 agonist, RO5166017. In the hippocampal CA1, bath application of ulotaront enhanced the field excitatory postsynaptic potential upon electrical stimulation of the Schaffer collateral pathway, while decreasing the spontaneous firing rate of glutamatergic pyramidal cells. The ability of ulotaront to selectively potentiate evoked synaptic responses, while reducing spontaneous activity suggests state-dependent modulation of glutamatergic tone. This observation is consistent with previous studies demonstrating state-dependent modulation of dopamine-synthesis capacity and MSN neural ensemble dynamics by ulotaront [33, 34].

Schizophrenia is a highly complex and heterogeneous disorder, and although multiple mechanistic hypotheses have been proposed (e.g., dopaminergic and glutamatergic dysfunction, genetic factors, neurodevelopmental alterations, etc,) no single hypothesis can fully explain all aspects of its etiology [5, 9]. Despite the complexity of the disease, current drug treatments still primarily rely on one mechanism of action: dopamine D_2_ receptor blockade. However, due to the significant side effects and often limited effectiveness [14–17], there is substantial interest in mechanistically novel and therapeutically more effective treatment approaches. TAAR1 agonism has been proposed as a new treatment strategy for schizophrenia and related neuropsychiatric disorders. The ability to impact the interaction between glutamatergic and dopaminergic circuits may underlie the beneficial behavioral effects of TAAR1 agonists in rodent models of psychiatric disorders [22, 25, 35]. This is supported by slice electrophysiology experiments showing that TAAR1 agonists can decrease VTA neuronal firing not only through direct action on the intrinsic excitability of dopamine neurons, but also by reducing glutamatergic, excitatory inputs to those cells [21, 23, 25]. TAAR1-mediated modulation of cortical glutamatergic transmission has also been of particular interest as its deficiency has been increasingly documented in a range of neuropsychiatric disorders including schizophrenia [6, 7, 24]. In fact, cortical glutamatergic projections to the midbrain have been shown to regulate the activity of mesostriatal dopamine neurons [36]. Other studies suggest that direct pathways from the prefrontal cortex to the midbrain exert an excitatory influence and enhance dopamine release, whereas indirect pathways involving GABAergic interneurons have the opposite effect [37].

Ulotaront is currently in Phase III clinical development following positive results in a randomized, placebo-controlled Phase II clinical trial in patients with an acute exacerbation of schizophrenia [30]. In addition to demonstrating efficacy, treatment with ulotaront was not associated with extrapyramidal side effects or metabolic disturbances, consistent with its novel mechanism of action and absence of D_2_ receptor blockade [30, 38, 39]. In contrast to conventional, target-driven strategies, ulotaront was discovered through a target-agnostic approach optimized to identify drug candidates that demonstrate a phenotypic antipsychotic-like profile in vivo but lack D_2_ and 5-HT_2A_ receptor antagonism [28]. Follow-up in vitro profiling showed that ulotaront is a full agonist at TAAR1 but also exhibits agonist activity at 5-HT_1A_ receptors [28]. More dedicated pharmacology studies determined that TAAR1 agonism contributes to ulotaront’s mechanism of action [28, 29]. Consistent with other TAAR1 agonists, ulotaront demonstrates antipsychotic-like efficacy in a broad range of rodent models and assays, including psychostimulant-induced abnormalities in locomotor activity, sensorimotor gating, social interaction and object recognition memory [28, 29, 40, 41]. Notably, ulotaront was reported to improve prepulse inhibition deficits induced by the glutamate NMDA receptor antagonist MK-801 in wild-type, but not TAAR1 knockout mice, suggesting that reversal of the effects are TAAR1-dependent [29]. Our data now indicate that ulotaront modulates excitatory glutamatergic synaptic transmission as well as intrinsic neuronal excitability in the hippocampus (where the principal neurons are glutamatergic cells), which is believed to play a central role in the pathophysiology for schizophrenia [42, 43]. These alterations in hippocampal activity might lead to downstream effects on dopamine neuron activity in the midbrain and striatum, complementing and/or supporting the previously reported capacity of ulotaront to decrease VTA neuronal firing and normalize elevated striatal dopamine synthesis capacity [28, 33]. In addition, effects on the glutamatergic system within the hippocampus and the cortex (including cortico-striatal projections) may be of relevance to negative and cognitive symptoms in schizophrenia, which have been associated with glutamate dysregulation in these brain regions. In this regard, TAAR1 has been shown to regulate cortical glutamate NMDA receptor function [24]. Our findings demonstrate prominent regulation of striatal and hippocampal excitatory neurotransmission by TAAR1 agonists and provide novel insight into TAAR1-mediated regulation of glutamatergic systems. Additionally, since we observed a modulation of the fEPSP peak amplitude (a component that reflects the integration of both excitatory and inhibitory inputs) in the CA1, future studies should elucidate potential, modulatory effects of uloratont on GABAergic synaptic activity.

A key hypothesis that links dopamine dysfunction to psychosis centers around aberrant salience attribution. It proposes that striatal dopamine dysregulation results in over-attribution of salience (i.e., significance or meaning) to neutral, or otherwise irrelevant, stimuli [44, 45]. Based on current and prior results, ulotaront might not only be able to regulate dopaminergic tone, but also modulate glutamatergic neurotransmission in the striatum. By reducing the basal glutamatergic activity (background noise) and amplifying the cortical-input-elicited excitatory signal in a cell type-specific manner, ulotaront may have the potential to improve information processing by selectively addressing the aberrant SNR reported in schizophrenic subjects; this mechanism might be alternative to the more classical role of dopamine in SNR optimization [46, 47]. This seemingly conflicting effect on the glutamatergic system (increase in evEPSC amplitude but decrease in mEPSC frequency) is in line with cumulative evidence suggesting that spontaneous and evoked synaptic activity might be independent pathways for neuronal communication that can be differentially modulated [48–50]. Interestingly, ulotaront produced a clear effect in putative D2-expressing MSNs, while only a non-significant trend was observed in D1-expressing MSNs, reinforcing the pivotal role of the former striatal subpopulation. A recent study analyzing neuronal activity in freely behaving animals also showed that the ulotaront-mediated modulation of activity level is more prominent in D2 rather than D1 MSNs [34]. Although additional studies are needed to address the TAAR1-dependance of ulotaront’s effects, it is notable that the selective TAAR1 agonist, RO5166017, not only produced the same cell type-specific bidirectional modulation of glutamatergic neurotransmission, but also elicited similar effect sizes and impacted the same proportion of cells. Further investigation is needed to elucidate whether these distinct subpopulations, with differential pharmacological responses, also display divergent efferent projections and/or molecular profiles. For example, cell-specific expression of G-protein-gated inwardly rectifying potassium (GIRK) channel subunits were reported to underly bidirectional effects of GABA_B_ receptor (a GPCR) agonists on the mesolimbic dopamine system [51]. Moreover, it remains to be determined whether the impact of ulotaront on striatal glutamatergic neurotransmission is a downstream effect of its modulation of dopaminergic signaling or if these two paths run in parallel and are finally integrated by MSNs.

MSNs are the principal computational units in striatum; their activity is driven by glutamatergic inputs from various cortical regions as well as the thalamus, and they project the output signal to several brain regions, including substantia nigra and globus pallidus [31]. By analyzing the mPSCs, we showed that ulotaront affects the excitatory but not the inhibitory synaptic activity, which suggests a modulatory effect on striatal E/I balance. Maintenance of the balance of excitatory and inhibitory signals is critical for the function of neural networks, and disruption of this finely tuned E/I balance has been proposed as part of a circuit-level mechanism for schizophrenia [6, 7, 52]. Since E/I balance is a multidimensional concept, additional approaches, including evaluation of ulotaront’s effects on stimulation-driven synaptic activity, are needed to expand the current findings. Whether TAAR1 agonism is solely responsible for ulotaront’s potential effects on E/I regulation, or whether 5-HT_1A_ agonism contributes to it, needs to be addressed. However, the selective TAAR1 agonist RO5166017 has been reported to impact pre- (glutamate release) and post-synaptic (phosphorylation of AMPA receptors) glutamatergic neurotransmission in the striatum of a Parkinson’s disease mouse model [35]. Furthermore, our results suggest that modulation of spontaneous excitatory synaptic activity likely rely on pre-synaptic mechanisms. Future studies will need to elucidate whether the regulation of spontaneous glutamatergic neurotransmission preferentially takes place at thalamic vs cortical presynaptic inputs or reflects a general modulation. In terms of the underlying molecular mechanisms, one potential hypothesis is that the observed effect on mEPSC frequency relies on a presynaptic interaction between TAAR1-D_2_ receptors, which has previously been shown in vitro [22]. Dopamine D_2_ receptors are located on pre and postsynaptic terminals, while D_1_ receptors are mainly found on postsynaptic elements. However, this is not easily reconciled with ulotaront’s preferential effect on postsynaptic D_2_-expressing neurons, suggesting involvement of other mechanisms. Since random fluctuations of the synaptic vesicle fusion machinery are sensitive to intracellular calcium levels [53], an alternative mechanistic scenario is that ulotaront regulates mEPSC frequency through TAAR1-mediated control of intracellular calcium [54]. More insight into the cellular and subcellular localization of TAAR1 is needed, something that has proven remarkably challenging due to the low expression levels in the brain and lack of reliable antibodies.

In combination with existing evidence, the current data suggest that TAAR1 agonists, including ulotaront, exert state-specific effects on dopaminergic and glutamatergic neurotransmission, synthesis and/or neuronal firing, which may serve to improve the altered signal-to-noise ratio and presynaptic dopamine dysfunction associated with psychosis. The impact of TAAR1 on glutamate-dopamine circuits across multiple disease-relevant brain regions speaks to the potential of this target not only for schizophrenia but also for CNS disorders in general.

### Supplementary information

Supplementary Information
