# Supplementary material for: TAAR1 agonist ulotaront modulates striatal and hippocampal glutamate function in a state-dependent manner
Source: Neuropsychopharmacology. 2023 Dec 19;49(7):1091–103. doi: 10.1038/s41386-023-01779-x (PMC11109157; doi:10.1038/s41386-023-01779-x)
Supplement: Supplementary file 1 — Supplementary Information [file 41386_2023_1779_MOESM1_ESM.docx]

**SUPPLEMENTARY INFORMATION** for

**TAAR1 Agonist Ulotaront Modulates Striatal and Hippocampal Glutamate Function in a State-Dependent Manner**

Sung M. Yang^1^, Ayan Ghoshal^1^, Jeffrey Hubbard^2^, Florian Gackière^2^, Romain Teyssié^2^, Stuart A. Neale^3^, Seth C. Hopkins^1^, Kenneth S. Koblan^1^, Linda J. Bristow^1^ and Nina Dedic^1,*^

^1^Sumitomo Pharma America, Inc., Marlborough, MA, USA

^2^Neuroservices Alliance, Le Puy Sainte Réparade, France

^3^Neurexpert Limited, Newcastle, UK

^*^Corresponding author; e-mail: Nina.Dedic@us.sumitomo-pharma.com

This PDF file includes:

- Supplementary Materials and Methods
- Supplementary Figures 1 to 5
- Table S1

**Supplementary Materials and Methods**

Animals

For recordings in the striatum, 8- to 12-week-old male Drd1a-tdTomato mice were provided by Charles River Laboratories, France (imported from Jackson Laboratories, mouse line #016204, USA). This mouse line expresses the reporter gene tdTomato under the control of the Drd1a promoter. For recordings in the hippocampal CA1 region, experiments were conducted with 6- to 8-week-old C57Bl6/J male mice (from Elevage Janvier, FRANCE or Charles River, UK).

Animals were group-housed and maintained in a room with controlled temperature (20-24°C) and a light-dark cycle (12h/12h; lights on: 7 am; lights off: 7 pm) with food and water available ad libitum. All procedures were designed to minimize animal suffering and performed in accordance with institutional and federal guidelines. Animals used in the striatal experiments and hippocampal CA1 MEA recordings were treated according to the European guidelines 2010/63/UE. The standard protocol describing the animal model used in this study was approved by the Animal Ethical Committee (Comité d'Ethique 71 pour l'Expérimentation Animale “Laurent Vinay” - CE71 from Neuroscience Institute of La Timone) and accredited by the French Ministry of Education and Research (MESR) under national authorization number #13288-2018022522441658 v5. Animals used for the hippocampal glass pipette firing and fEPSP recordings were treated in accordance with the experimental conditions and procedures of the UK Animals (Scientific Procedures) Act 1986 and associated guidelines, and in compliance with the ARRIVE guidelines.

Slice preparation

***Striatum recordings.*** Mice were deeply anesthetized using isoflurane at 2.5% for a 4-minute induction and at ~2% for maintenance to perform transcardiac perfusion with cold (2-4°C) sucrose solution containing (in mM): sucrose 248, D-glucose 11, NaHCO_3_ 26, KCl 2, NaH_2_PO_4_ 1.25 and MgSO_4_ 2. The brain was extracted and plunged in ice-cold oxygenated sucrose solution for cutting. Acute coronal brain slices (300 μm thickness) were prepared with a VT1200S vibratome (Leica Biosystems). After cutting, about 6 slices containing the striatum were collected in an interface chamber (Brain Slice Keeper 5, Scientific Systems Design) and left to recover during 10 minutes at 32-34°C in a solution containing (in mM): NMDG 98, D-glucose 25, NaHCO_3_ 30, HEPES 20, Na/L-Ascorbate 5, ethyl pyruvate 2, Thiourea 2, KCl 2.5, NaH_2_PO_4_ 1.25, MgSO_4_ 10, CaCl_2_ 0.5 and N-acetyl-L-cysteine 12. Recovery was individually timed for each slice to ensure precise timing of this step. Slices were then moved to a second interface chamber and kept at least 1 hour in room temperature aCSF (artificial Cerebro-Spinal Fluid) with the following composition (in mM): NaCl 90, D-glucose 25, NaHCO_3_ 30, HEPES 20, Na/L-Ascorbate 5, ethyl pyruvate 2, Thiourea 2, KCl 2.5, NaH_2_PO_4_ 1.25, MgSO_4_ 2 and CaCl_2_ 2.5.

***Hippocampus recordings with MEA.*** Mice were sacrificed by fast decapitation, without anesthesia. The brain was quickly removed and soaked in ice-cold (1–4 °C) NMDG-based cutting solution with the following composition (in mM): NMDG 93, KCl 2.5, NaH_2_PO_4_ 1.2, NaHCO_3_ 25, HEPES 20, glucose 25, thiourea 2, Na-ascorbate 5, Na-pyruvate 3, CaCl_2_ 0.5 and MgSO_4_ 10, and pH titrated to 7.3–7.4 with concentrated hydrochloric acid. Coronal slices (300 μm thickness) containing the hippocampus were prepared with a Leica VT1200S vibratome. Slices were placed for 15 minutes in an interface chamber containing NMDG-base cutting solution (at 32°C), and then let to recover at 32°C for 60 minutes in aCSF with the following composition (in mM): NaCl 126, KCl 3.5, NaH_2_PO_4_ 1.2, MgCl_2_ 1.3, CaCl_2_ 2, NaHCO_3_ 25 and glucose 11.

***Hippocampus recordings with glass pipette.*** Mice were anesthetized with inhaled isoflurane and then killed by cervical dislocation. The brain was removed and placed into ice-cold oxygenated sucrose medium containing (in mM): 252 sucrose, 3 KCl, 1.25 NaH_2_PO_4_, 1 MgSO_4_, 1.2 CaCl_2_, 10 glucose and 24 NaHCO_3_. The brain was hemisected along the midline and 400-µm parasagittal slices were prepared with an oscillating microtome (Campden Instruments Ltd., Loughborough, UK). Slices were then transferred to a recovery chamber maintained at room temperature and containing oxygenated solution with the following composition (in mM): 126 NaCl, 3 KCl, 1.25 NaH_2_PO_4_, 1 MgSO_4_, 1.2 CaCl_2_, 10 glucose and 24 NaHCO_3._ Slices were then maintained at room temperature for at least 60 minutes before recording.

All solutions were continuously bubbled with carbogen gas (95% O_2_/5% CO_2_) to maintain a high level of oxygenation, and adjusted to pH = 7.3-7.4 and 305-310 mosmol/L.

Compound preparation

Ulotaront HCl (SEP-363856, MW free base = 183.27 g/mol; MW salt = 219.73 g/mol) and RO5166017 (MW free base = 219.28 g/mol) were synthesized by Sumitomo Pharma America Inc. Both compounds were freshly prepared on each recording day as a stock solution in deionized water, and then adequately diluted in aCSF to reach the final test concentration.

Patch clamp recordings

***Striatum recordings.*** Slices were transferred to a recording chamber continually perfused at a rate of 3-5 mL/min with recording aCSF containing (in mM): NaCl 126, KCl 3.5, CaCl_2_ 2, MgCl_2_ 1.3, NaHCO_3_ 25, NaH_2_PO_4_ 1.2 and D-glucose 11. Bath temperature was controlled by a bath heater apparatus (Scientifica) set to 25 ± 1°C (miniature PSC) or a flow heater device (Scientifica) set to 30 ± 1°C (evEPSC). Whole-cell patch-clamp experiments were performed in voltage-clamp mode using a software-controlled MultiClamp 700B amplifier and a Digidata 1440A digitizer (all from Molecular Devices). Data were low-pass-filtered at 2 kHz and sampled at 10 kHz using Clampex 10 software (Molecular Devices). Patch pipettes were pulled from borosilicate glass capillaries (1.5 mm outer diameter, World Precision Instruments TW150F-4) with a resistance of 3-5 MΩ. Recordings of synaptic activity were performed in the dorsal striatum.

*Miniature postsynaptic current.* The internal solution to record miniature excitatory postsynaptic current (mEPSC; holding potential V_hold_ = -80 mV) was composed of (in mM): 105 K-gluconate, 30 KCl, 10 HEPES, 0.3 EGTA, 4 MgCl_2_, 4 Na-ATP, 0.3 Na-GTP and 10 Na-phosphocreatine. The internal solution to record miniature inhibitory post-synaptic current (mIPSC; V_hold_ = -65 mV) contained (in mM): 140 CsCl, 10 NaCl, 1 EGTA, 10 HEPES and 0.1 CaCl_2_. Both internal solutions were adjusted to 285-295 mosmol/L and pH = 7.3 using KOH.

After a period of stabilization (3-5 min), miniature postsynaptic currents were recorded in the presence of 0.5 μM tetrodotoxin (TTX). mEPSCs and mIPSCs were isolated in the presence of 50 μM picrotoxin (PTX) and 30 μM D-AP5 + 10 μM NBQX, respectively. Occasionally, 30 μM D-AP5 + 10 μM NBQX or 50 μM PTX were applied at the end of the experiment; the treatment with these selective antagonists robustly abolished the mEPSCs and mIPSCs, respectively, confirming their glutamatergic and GABAergic nature. Miniature PSCs were recorded for a total duration of 20 min (5-min baseline followed by 15-min vehicle or compound perfusion), interspersed every 5 min by a test pulse protocol (digitized at 20 kHz) to monitor series resistance (Rs).

*Evoked postsynaptic currents.* The internal solution to record evoked excitatory postsynaptic currents (evEPSC) was composed of (in mM): 105 K-gluconate, 30 KCl, 10 HEPES, 0.3 EGTA, 4 MgCl_2_, 4 Na-ATP, 0.3 Na-GTP and 10 Na-phosphocreatine or 135 K-gluconate, 3 KCl, 10 HEPES, 0.5 EGTA, 3 MgCl_2_, 4 Na-ATP, 0.3 Na-GTP and 10 Na-phosphocreatine (adjusted to pH = 7.3, 285-295 mosmol/L). To record the evEPSCs, V_hold_ was set at -80 mV.

Electrical stimulation was performed using a tungsten bipolar concentric (FHC, CBBSE75) or tungsten bipolar (home-made) electrode placed in the deep cortical layer, close to the white matter (corpus callosum). Duration (0.1-0.2 msec) and intensity (1-5 mA) were adjusted for each neuron; the intensity of stimulation was set to elicit ~40% of the maximal evEPSCs amplitude (~40% Imax). Each sweep of the protocol, delivered at a frequency of 0.05 Hz, integrated a dual stimulation (paired-pulse stimulation for paired-pulse ratio (PPR) evaluation; interstimulus interval of 50 msec) to elicit the evEPSC as well as a test pulse (digitized at 2 kHz) to monitor series resistance (Rs).

Evoked-EPSCs were recorded in the presence of 20 µM bicuculline to block GABA_A_ receptors. After 5 min of baseline recording in vehicle (aCSF), the compound of interest or vehicle were bath perfused for a total duration of 15 min. Occasionally, 10 μM NBQX was applied at the end of the experiment; this treatment robustly abolished the evEPSCs, confirming their glutamatergic nature.

*Striatal MSN identification.* Neurons were visualized using infrared differential interference contrast microscopy (IR-DIC) and 60x objective. TdTom^+^ (D1-receptor-expressing or D1-positive) and tdTom^-^ (D1-receptor-non-expressing or D1-negative, putative D2-receptor-expressing) MSNs in the dorsal striatum were identified by combining the fluorescence and IR-DIC images, and then visually targeted for whole-cell recording. Images of the recorded neurons were taken to confirm the presence or absence of tdTomato expression.

Striatal medium spiny neurons were identified by their morphology and their electrophysiological properties (assessed in current-clamp mode): 1) hyperpolarized resting membrane potential (RMP; around -80 mV); 2) delayed and regular firing properties upon depolarizing current step injections; and 3) the presence of a voltage “sag” in response to hyperpolarizing current step injections, due to activation of HCN channels. Because mIPSCs were recorded using a cesium-based internal solution and CsCl blocks the HCN channels, the identification of MSNs by current step injections was performed immediately after breaking into whole-cell configuration. This identification was confirmed by the presence of a voltage “sag” in response to depolarizing current step injections due to delayed firing, typical of MSNs.

*Inclusion criteria.* A list of inclusion criteria was applied to validate the recordings, namely: MSN electrophysiological properties, D1^+^ or D1^-^ fluorescence, Rs under 25 MΩ, Rs variation < ± 20% (only when correlated with an inverse change in evEPSC amplitude), holding current < 300 pA, and stability during the baseline period (assessed by a linear regression with a p-value > 0.1).

Spontaneous firing activity

***MEA multi-unit recordings in hippocampus.*** All data were recorded with a multi-electrode array (MEA) setup commercially available from Multichannel Systems (MCS GmbH, Reutlingen, Germany), composed of a 4-channel stimulus generator and a 60-channel amplifier head-stage connected to a 60-channel A/D card. Software for recording and analysis was the one commercially available from Multichannel Systems: MC Rack. The MEAs consisted of 60 electrodes spaced by 100 µm. Firing activity was recorded in continuous mode with a sampling rate set to 20 kHz.

During the recording, slices were continuously perfused at the rate of 3 mL/min with oxygenated aCSF at 32°C. One line of electrodes was centered on the CA1 pyramidal cells. Four conditions were recorded in parallel with the following experimental workflow: after a 20-minute baseline, ulotaront was applied for 40 minutes at 1 µM, 10 µM or 30 µM; then, ulotaront was washed out for 20 minutes, followed by 20 minutes of Diazepam (30 µM) application as positive assay control. At the end, 1 µM TTX was applied for 10 minutes to assess background noise.

***Micropipette single-unit recordings in hippocampus.*** Following at least 60 minutes of recovery from the slicing procedure, individual slices were transferred to an interface recording chamber where they were continuously perfused with recording solution containing (in mM): 126 NaCl, 3 KCl, 1.25 NaH_2_PO_4_, 1 MgSO_4_, 1.2 CaCl_2_, 10 glucose and 24 NaHCO_3_ (at 32-33°C). Extracellular field potential recordings were made with an amplifier (Axon Instruments Ltd., USA) via a glass micropipette (resistance ~5 MΩ; filled with recording solution) positioned in the stratum pyramidale of the CA1. The electrode was positioned using a microscope and a suitable recording position was selected based on the observation of clear single units in real time. Data were digitized (10 kHz) via a CED1401 interface and stored on a computer with Signal software (Cambridge Electronic Design Ltd., Cambridge, UK).

Following stabilization, spontaneous firing activity was recorded for 10 min after which ulotaront (30 μM) was applied to the bath for 20 min and effects on neuronal firing determined. For comparison, parallel recordings were made in a separate set of slices exposed to application of vehicle. On completion of testing, all slices were exposed for 15 min to normal recording solution to facilitate the washout, followed by 20 min in 1 μM tetrodotoxin (TTX) to validate that the recorded units were action potentials.

Field potential recordings

***Field EPSP in hippocampus.*** Following at least 60 minutes of recovery from the slicing procedure, individual slices were transferred to an interface recording chamber where they were continuously perfused with recording solution containing (in mM): 126 NaCl, 3 KCl, 1.25 NaH2PO4, 1 MgSO4, 1.2 CaCl2, 10 glucose and 24 NaHCO3 (at 32-33°C). Extracellular field potential recordings were made with an amplifier (Axon Instruments Ltd., USA) via a glass micropipette (resistance ~5 MΩ; filled with recording solution) positioned in the stratum radiatum of the CA1, digitized (10 kHz) via a CED1401 interface and stored on a computer with Signal software (Cambridge Electronic Design Ltd., Cambridge, UK). Field excitatory post-synaptic potential (fEPSP) responses were evoked by a bipolar stimulation electrode positioned in the stratum radiatum near the CA3-CA1 border. The stimulation electrode was used to deliver a pair of 0.02-ms pulses, separated by 40 ms, and applied every 10 s; the intensity of stimulation was adjusted to elicit approximately 60% of the maximal spike-free response.

Following stabilization, fEPSP responses were recorded for 10 min after which ulotaront was applied to the bath for 20 min (each concentration in different set of slices) and effects on synaptic transmission determined; ulotaront was tested at 10 and 30 μM. For comparison, parallel recordings were made in a separate set of slices exposed to application of vehicle. On completion of testing, all slices were exposed for 10 min to normal recording solution to facilitate the washout.

Analysis of electrophysiological recordings

***Miniature postsynaptic currents.*** Raw traces were offline filtered using a 1-kHz lowpass Bessel filter. The mPSCs were automatically detected using a custom-made script in Igor 6 (WaveMetrics). The frequency and amplitude of individual mPSCs were measured, averaged every 1 minute, and expressed as a function of time for each individual neuron. Frequency and amplitude of mPSC were also averaged using 5-min windows for further analysis: the last 5 min of baseline recording (from -5 to 0 min) was compared to 15 min after vehicle or compound perfusion (average of 10 to 15 min).

For the variance analysis, the coefficient of variation (CV^2^) and the variance-to-mean ratio (VMR) were calculated from the amplitude of individual mPSCs. They are defined as: ${CV}^{2}=\frac{\sigma^{2}}{\mu^{2}}$ and $VMR=\frac{\sigma^{2}}{\mu}$, where μ is the mean amplitude of the postsynaptic response and $\sigma^{2}$ is its variance. Quantal model of synaptic transmission suggests that CV^2^ depends on N (number of functional vesicle release sites) and Pr (probability of vesicle release) but is independent of Q (quantal size), while VMR is dependent on Pr and Q, but not on N. Thus, for example, a change in CV^2^ and VMR would be in line with the hypothesis that presynaptic changes (i.e, probability of vesicle release) are driving the effect on mPSCs.

***Evoked postsynaptic currents.*** The analysis was performed using Clampfit software to measure the peak amplitude as well as the rise time and decay time of evoked responses. The paired stimulation elicited two time-locked responses; only the kinetics of the first evoked response was analyzed. To analyze the evEPSC parameters, the average was calculated using 1-min bins (3 data points at 0.05 Hz sampling rate). Then, the evEPSC parameters throughout the recording session are presented as time courses and scatter plots (average of the last 3 min of each liquid period; baseline, from -3 min to 0 min, and treatment, from 12 min to 15 min); normalization was performed using the 5 first minutes of baseline period.

The normalized-to-baseline evEPSC amplitude was fit by a series of Gaussian mixture models, with 1 to 4 components (Gaussian mixture model is a probabilistic model that assumes all the data points are generated from a mixture of a finite number of Gaussian distributions, or components, with unknown parameters). Mixture models don’t require knowing which subpopulation a data point belongs to, instead it allows the model to learn the subpopulation automatically. Minimization of the Bayes information criterion (BIC) was used to identify the optimal number of components in an unsupervised manner (Fig. 3G; BIC is a criterion for model selection among a finite set of models). Post-fitting, since subpopulation assignment is not known, data was partitioned according to the fit component with the largest posterior probability, weighted by the component probability. All analysis was done in Matlab, using Statistics Toolbox version 12.2 (fitgmdist.m and cluster.m).

***MEA multi-unit recordings.*** The raw data were filtered with a high-pass filter (Butterworth second order filter, set at 200 Hz). The threshold for detecting spikes was -4 times the standard deviation of the baseline noise, and a dead time of 2 ms was applied after each detected spike before starting the search for the next spike. The firing rate was calculated as the spike number per second recorded at each electrode and it was averaged using 30-s bins. Only the electrodes displaying a steady firing rate greater than 0.5 Hz and stable over the 20-minute baseline were validated (less than 15% of variation). For an electrode to be selected, it also needed to display at least 90% of firing rate inhibition in the presence of 1 µM TTX.

***Micropipette single-unit recordings.*** The recordings were analysed offline with a spike sorting algorithm (supplied with Spike2, Cambridge Electronic Design, UK) to identify the action potentials, and then spike frequency was quantified in consecutive fixed 1-second bins. In each recording, firing rate was normalised to the mean of 10-minute data collected prior to application of ulotaront or vehicle. Then, the average of 5-min consecutive acquisition was calculated: 1) prior to ulotaront or vehicle application (baseline, from 5 min to 10 min), and 2) at the end of 20-minute application of ulotaront or vehicle (treatment, from 25 min to 30 min). The mean values for each phase were used for analysis and comparison. At the end of each recording TTX (1 µM) was applied to demonstrate compound access to the slice. Data were first assessed for outliers (ROUT method (Q=1%) within Graphpad Prism) – note that the raw frequency rate was not assessed for outliers as cell-to-cell variability is expected. If a single outlier was identified in any of the analysis periods (baseline or treatment), then all values for that unit were excluded from that analysis set.

***Field EPSP.*** Recordings were assessed for effects on initial fEPSP slope, fEPSP peak amplitude and paired-pulse ratio, a form of presynaptic plasticity. In each recording, measures were normalized to the mean of 10-minute data collected prior to application of ulotaront or vehicle. Then, the average of 5-min consecutive acquisition was calculated: 1) prior to ulotaront or vehicle application (baseline), and 2) at the end of 20-minute application of ulotaront or vehicle (treatment).

The fEPSP response elicited by the 1st pulse of each pair of stimuli (i.e., fEPSP1) was used to determine effects on fEPSP amplitude and initial slope. The paired-pulse ratio was defined as the ratio of the fEPSP amplitude of the 2nd pulse (i.e., fEPSP2) to that of the first one (i.e., PPR = fEPSP2/fEPSP1).

Data presentation and statistical analysis

To assess compound effects, two different approaches were applied. Firstly, two-way ANOVA with repeated measures (followed by Bonferroni’s multiple comparison tests across time, within compound) was applied on the raw data, using the “time factor” (baseline vs post-compound) and the “compound factor” (vehicle vs ulotaront). Secondly, normalized-to-baseline data was used to evaluate unpaired comparisons (unpaired t-test, Mann Whitney test or one-way ANOVA) between the vehicle-treated and compound-treated groups.

Table S1 contains the statistical tests used to measure significance, the corresponding significance level (P value) and sample size. Normality was assessed using Shapiro-Wilk’s test or D'Agostino-Pearson normality test at a p-value of 0.05. When a data set did not satisfy normality criteria, nonparametric statistics were applied. Two-tailed Mann-Whitney U test was used for single comparisons, and two-tailed Wilcoxon matched-pairs signed rank test was used for paired values. For normal distributions, homoscedasticity was assessed using Bartlett’s test and F-test, at a p-value of 0.05. For homogeneous variances, two-tailed t-test was used for single comparisons, and repeated-measures one-way ANOVA followed by post hoc Dunnett’s test was used for statistical analysis of time course data. Paired t-test was used to compare paired data. In the only case where variances were not homogeneous, a t-test with Welch’s correction was used. No statistical methods were used to predetermine sample sizes, but our sample sizes are similar to those reported in previous publications and consistent with those used in the field. Unless otherwise specified, data are presented as mean ± s.e.m. (text and figures). Statistical tests were performed using GraphPad Prism version 9.3.1 (GraphPad Software, Inc) or MATLAB 2021b (The MathWorks Inc., Natick, MA), and p < 0.05 was considered statistically significant. Results from statistical analysis were indicated by p-values: * < 0.05, ** < 0.01, *** < 0.001, **** < 0.0001; ns, non-significant.

**Supplementary Figures**


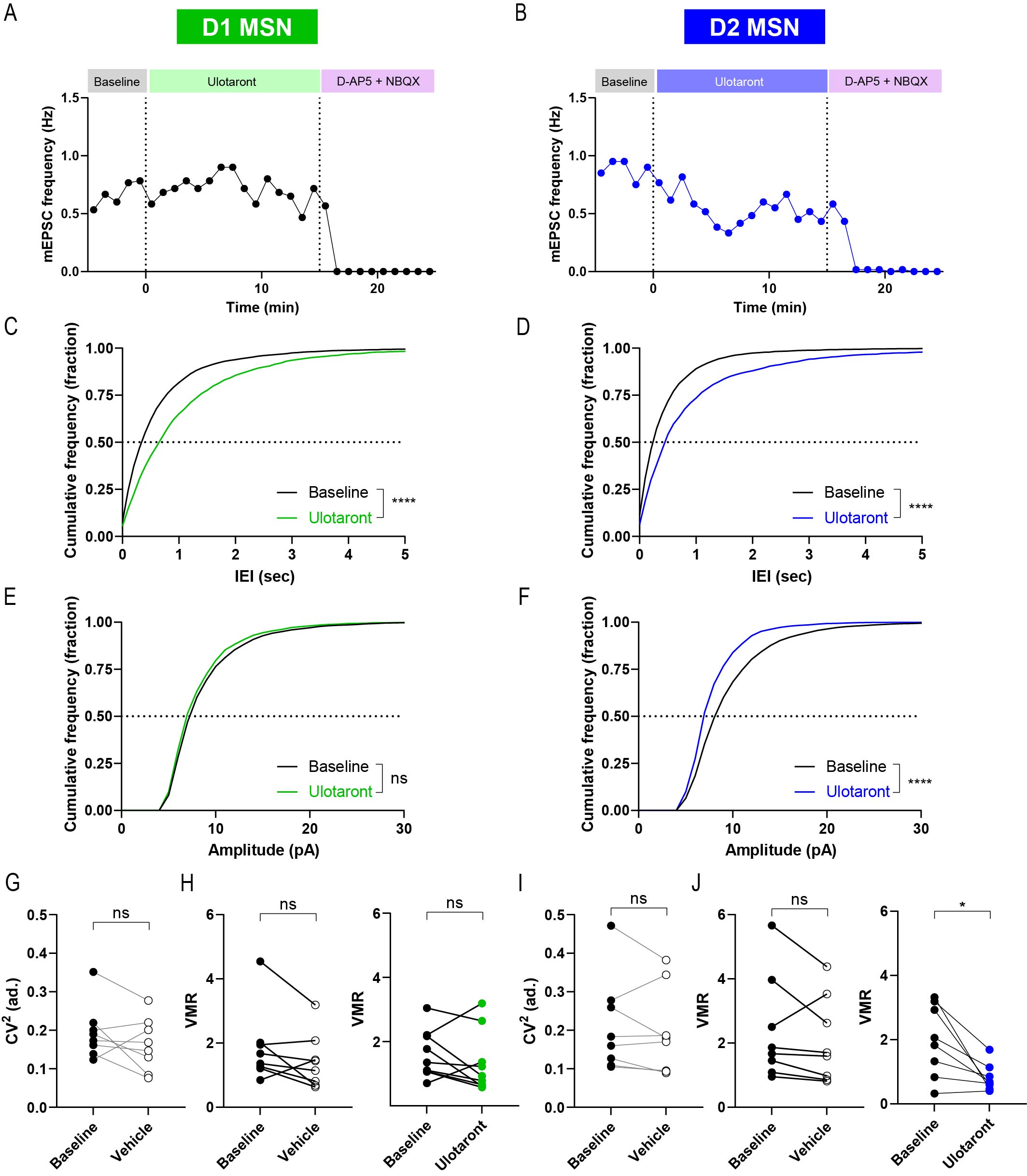


**Supplementary Figure 1.** **Effect of ulotaront on mEPSC in striatal MSNs. (A** to **B)** Example time courses of the mEPSC frequency in D1-expressing (**A**) and D1-non-expressing (putative D2-expressing; **B**) MSNs. At the end of the experiment, D-AP5+NBQX were perfused in the bath to validate that mEPSCs were glutamatergic responses. (**C** to **F**) Inter-event interval (IEI; **C**, **D**) and amplitude (**E**, **F**) of mEPSC in D1-expressing (left column) and putative D2-expressing (right column) MSNs, presented in cumulative frequency plots. (**G** to **J**) Analysis of coefficient of variation (CV^2^; **G**, **I**) and variance-to-mean ratio (VMR; **H**, **J**) applied on the mEPSC amplitude, comparing the baseline and treatment conditions for both the vehicle and ulotaront groups (CV^2^ for ulotaront is presented in Figure 1). In *G* to *J*, each dot represents a cell. Statistics are described in table S1. ns, not significant; * p<0.05; **** p<0.0001.


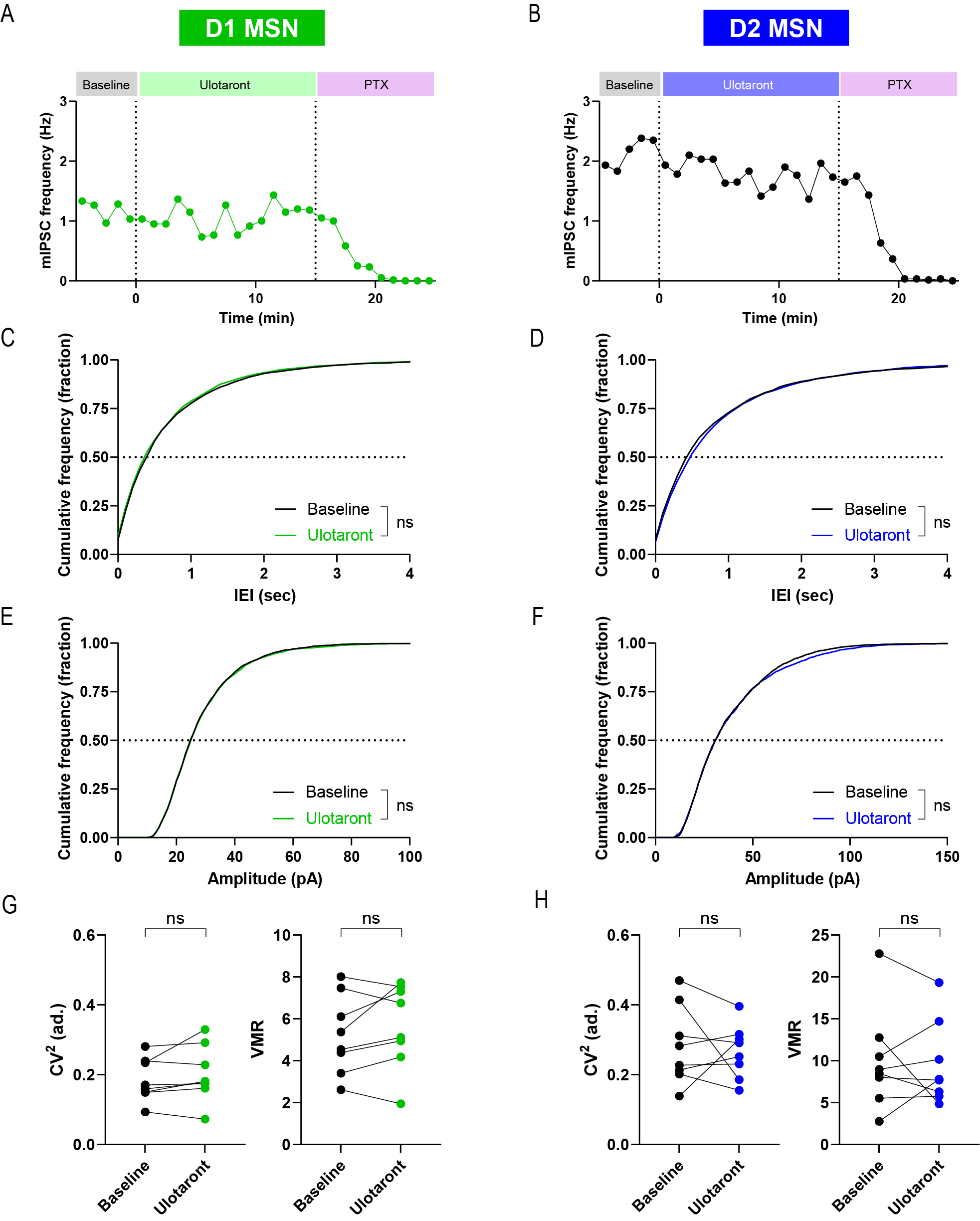


**Supplementary Figure 2.** **Effect of ulotaront on mIPSC in striatal MSNs. (A** to **B)** Example time courses of the mIPSC frequency in D1-expressing (**A**) and D1-non-expressing (putative D2-expressing; **B**) MSNs. At the end of the experiment, picrotoxin (PTX) was perfused in the bath to validate that mIPSCs were GABAergic responses. (**C** to **F**) Inter-event interval (IEI; **C**, **D**) and amplitude (**E**, **F**) of mIPSC in D1-expressing (left column) and putative D2-expressing (right column) MSNs, presented in cumulative frequency plots. (**G** to **H**) Coefficient of variation (CV^2^) and variance-to-mean ratio (VMR) analyses were applied on the mIPSC amplitude, comparing the baseline and ulotaront conditions. In *G* and *H*, each dot represents a cell. Statistics are described in table S1. ns, not significant.


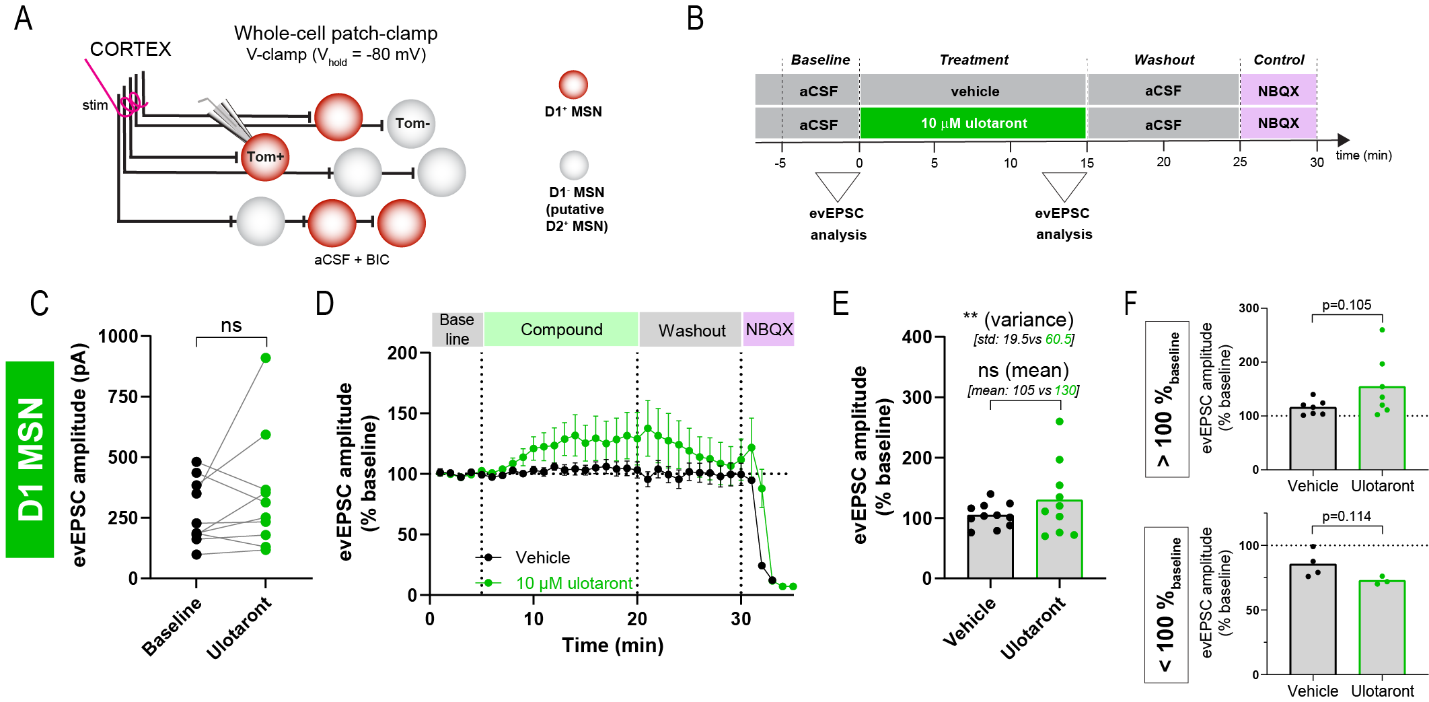


**Supplementary Figure 3.** **Effect of ulotaront on evEPSC in D1-expressing MSNs.** (**A** to **B**) Schematics of recording strategy (**A**) and experimental time course (**B**). Evoked EPSCs were elicited by stimulation of the deep cortical layer and recorded in D1-expressing (tdTom^+^) MSNs using whole-cell V-clamp configuration, with bicuculline (BIC) added into the aCSF. Evoked EPSC amplitude was analyzed during pre-drug baseline condition and for the last 3 minutes of treatment with 10 μM ulotaront or vehicle. (**C**) Amplitude during baseline condition and upon bath application of ulotaront. (**D**) Average time course of the normalized evEPSC amplitude. (**E** to **F**) Comparison of the normalized-to-baseline evEPSC amplitude for vehicle- vs ulotaront-treated group. The entire population was analyzed at the level of mean and variance (average mean and standard deviation are presented in parentheses; **E**). The data were also split in two groups based on the direction of the change relative to baseline (> baseline vs < baseline; **F**). In *C*, *E* and *F*, each dot represents a cell. Data are mean ± s.e.m. Statistics are described in table S1. ns, not significant; ** p<0.01.


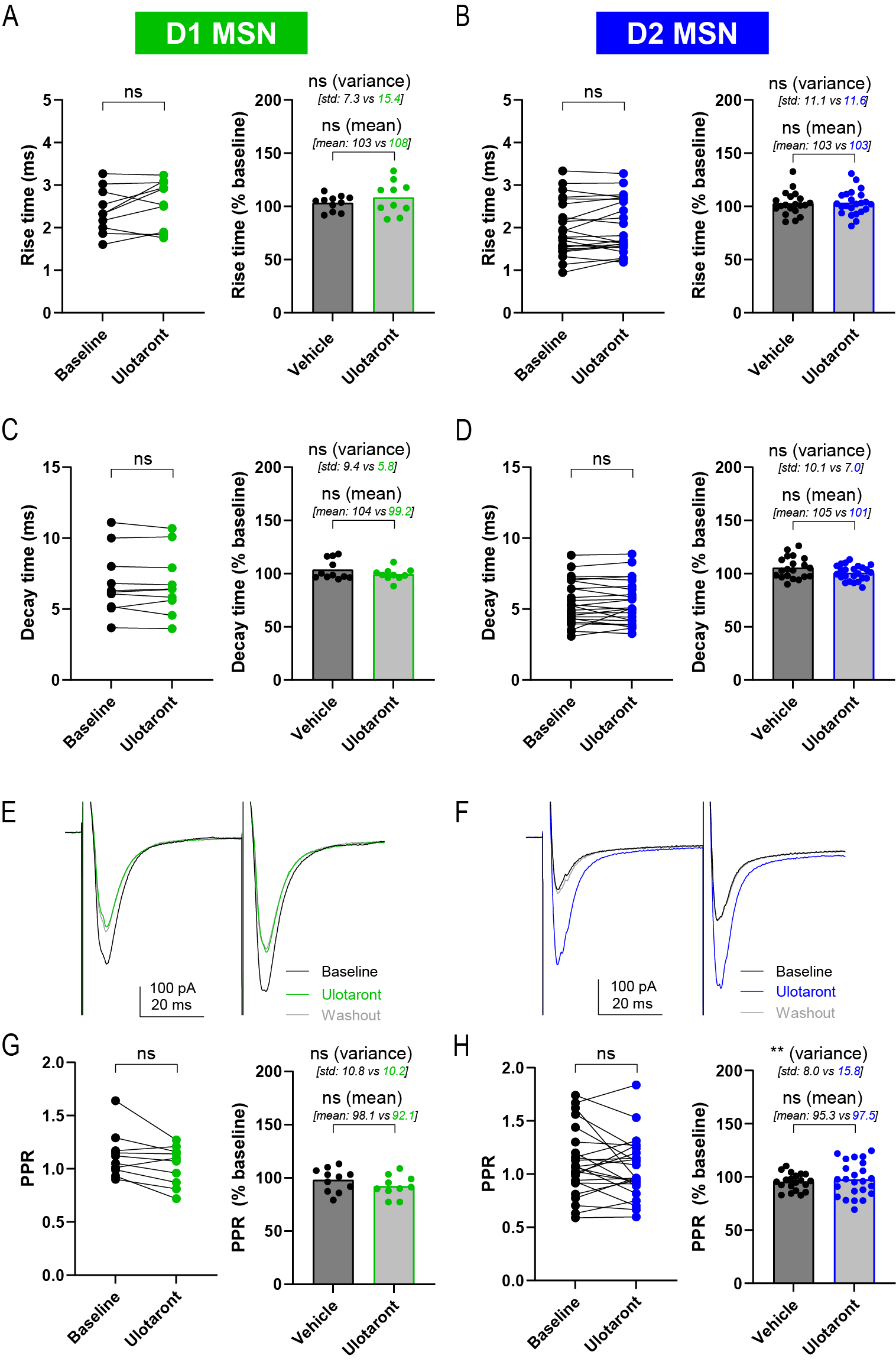


**Supplementary Figure 4.** **Ulotaront does not alter the kinetic properties and PPR of evEPSC in striatal MSNs.** (**A** to **D**) Kinetic properties of evEPSC in D1-expressing (**A** and **C**) and putative D2-expressing (**B** and **D**) MSNs. Effect of ulotaront application (10 μM) on rise time (**A** and **B**) and decay time (**C** and **D**) of evEPSC was compared against the baseline condition (left subpanel) and the vehicle group (after normalization to baseline; right subpanel). For the latter, the analysis was performed at the level of mean and variance (average mean and standard deviation are presented in parentheses). (**E** to **H**) Paired-pulse ratio of evEPSC amplitude in D1-expressing (**E** and **G**) and putative D2-expressing (**F** and **H**) MSNs. (**E** and **F**) Average representative traces of the evEPSC responses elicited by a pair of electrical pulse stimulation. (**G** and **H**) Effect of ulotaront application (10 μM) on PPR of evEPSC amplitude was evaluated by comparing against the baseline condition (left subpanel). Normalized-to-baseline PPR was analyzed by comparing vehicle and ulotaront groups (right subpanel) at the level of mean and variance (average mean and standard deviation are presented in parentheses). In *A* to *D* and *G* to *H*, each dot represents a cell, and bars represent the mean. Statistics are described in table S1. ns, not significant.


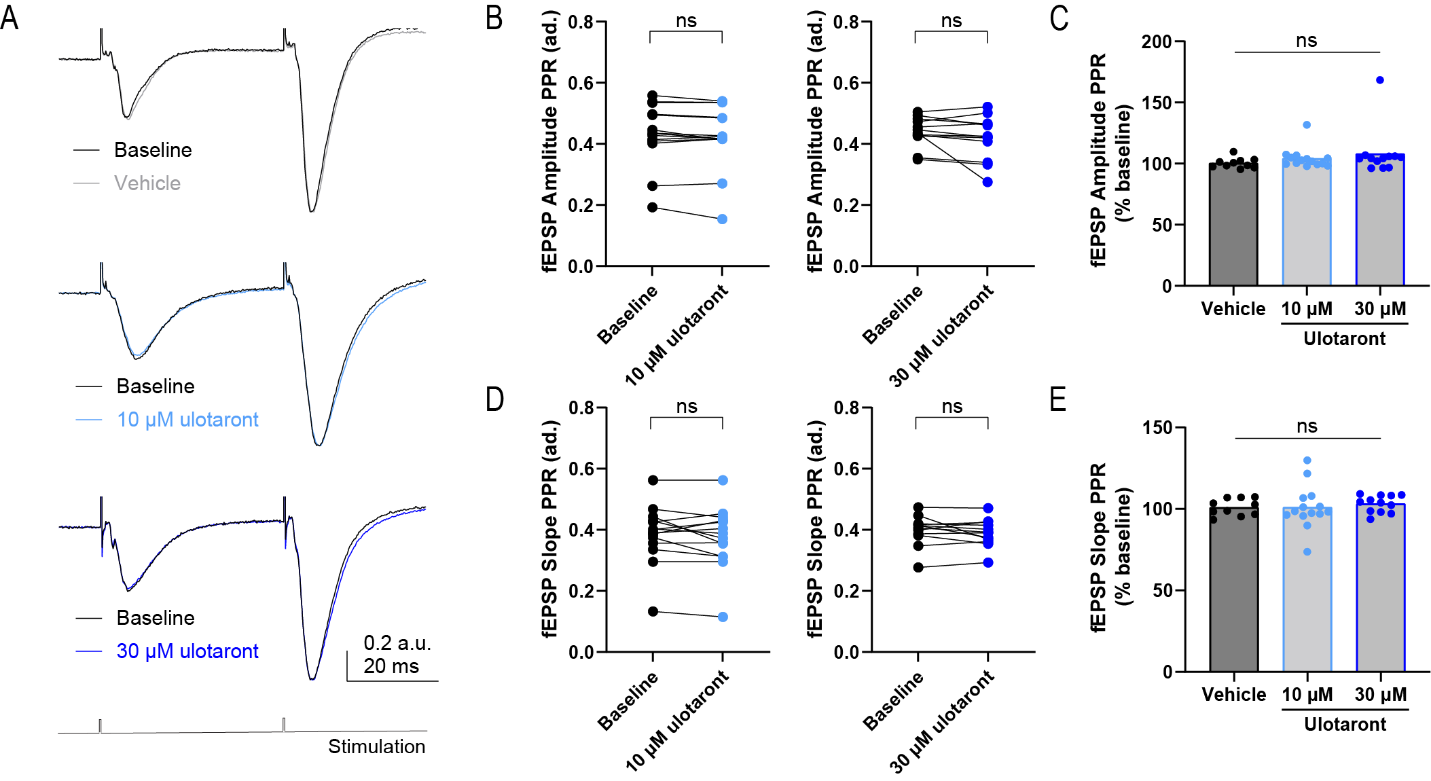


**Supplementary Figure 5.** **Ulotaront does not alter the paired-pulse ratio of fEPSP in the CA1.** (**A**) Representative average traces of fEPSP responses upon delivery of two pulses (40-ms separation) of electrical stimulation onto the Schaffer Collateral pathway. Traces were normalized by the peak amplitude of the fEPSP response elicited by the second pulse (i.e., the normalized amplitude of the first peak corresponds to the amplitude PPR). (**B** to **C**) Effect of ulotaront on PPR measured from the fEPSP amplitude was addressed by comparing against the baseline condition (**B**) and vs vehicle group (after normalization to baseline; **C**). (**D** to **E**) Evaluation of fEPSP slope PPR, corresponding to (B) to (C). In *B* to *E*, each dot represents a cell, and bars represent the mean. Statistics are described in table S1. ns, not significant.

Table S1. Statistical analysis

| **Figure** | **Sample Size** | **Statistical Test** | **Values** |
| --- | --- | --- | --- |
| **1D** | Vehicle: 8 cells  Ulotaront: 9 cells | Two-way RM ANOVA followed by Bonferroni’s multiple comparison tests (across time, within compound) | Subject: P = 0.0013, F (15, 15) = 5.293  Time: P = 0.0008, F (1, 15) = 17.33  Compound: P = 0.690, F (1, 15) = 0.166  Time x Compound: P = 0.273, F (1, 15) = 1.294  baseline vs treatment  Vehicle: P = 0.110  Ulotaront: P = 0.0031 |
| **1E, right** | Vehicle: 8 cells  Ulotaront: 9 cells | Unpaired t-test  (two-tailed) | P = 0.240  (t_15_ = 1.224) |
| **1F** | Vehicle: 8 cells  Ulotaront: 9 cells | Two-way RM ANOVA followed by Bonferroni’s multiple comparison tests (across time, within compound) | Subject: P < 0.0001, F (15, 15) = 19.55  Time: P = 0.0123, F (1, 15) = 8.102  Compound: P = 0.665, F (1, 15) = 0.1949  Time x Compound: P = 0.435, F (1, 15) = 0.6442  baseline vs treatment  Vehicle: P = 0.053  Ulotaront: P = 0.314 |
| **1G, right** | Vehicle: 8 cells  Ulotaront: 9 cells | Unpaired t-test  (two-tailed) | P = 0.466  (t_15_ = 0.749) |
| **1H** | 9 cells | Paired t-test (two-tailed) | P = 0.242  (t_8_ = 1.263) |
| **1J** | Vehicle: 8 cells  Ulotaront: 8 cells | Two-way RM ANOVA followed by Bonferroni’s multiple comparison tests (across time, within compound) | Subject: P < 0.0001, F (14, 14) = 11.98  Time: P < 0.0001, F (1, 14) = 37.15  Compound: P = 0.072, F (1, 14) = 3.79  Time x Compound: P = 0.0015, F (1, 14) = 15.40  baseline vs treatment  Vehicle: P = 0.110  Ulotaront: P = 0.0031 |
| **1K, right** | Vehicle: 8 cells  Ulotaront: 8 cells | Unpaired t-test  (two-tailed) | P = 0.006  (t_14_ = 3.235) |
| **1L** | Vehicle: 8 cells  Ulotaront: 8 cells | Two-way RM ANOVA followed by Bonferroni’s multiple comparison tests (across time, within compound) | Subject: P < 0.0001, F (14, 14) = 10.23  Time: P = 0.133, F (1, 14) = 2.545  Compound: P = 0.002, F (1, 14) = 13.93  Time x Compound: P = 0.064, F (1, 14) = 4.040  baseline vs treatment  Vehicle: P = 0.487  Ulotaront: P = 0.0023 |
| **1M, right** | Vehicle: 8 cells  Ulotaront: 8 cells | Unpaired t-test  (two-tailed) | P = 0.056  (t_14_ = 2.084) |
| **1N** | 8 cells | Paired t-test (two-tailed) | P = 0.011  (t_7_ = 3.413) |
| **2C** | Vehicle: 8 cells  Ulotaront: 8 cells | Two-way RM ANOVA followed by Bonferroni’s multiple comparison tests (across time, within compound) | Subject: P = 0.0001, F (14, 14) = 8.907  Time: P = 0.921, F (1, 14) = 0.010  Compound: P = 0.788, F (1, 14) = 0.075  Time x Compound: P = 0.303, F (1, 14) = 1.142  baseline vs treatment  Vehicle: P = 0.844  Ulotaront: P > 0.999 |
| **2D, right** | Vehicle: 8 cells  Ulotaront: 8 cells | Unpaired t-test  (two-tailed) | P = 0.188  (t_14_ = 1.383) |
| **2E** | Vehicle: 8 cells  Ulotaront: 8 cells | Two-way RM ANOVA followed by Bonferroni’s multiple comparison tests (across time, within compound) | Subject: P < 0.0001, F (14, 14) = 14.67  Time: P = 0.505, F (1, 14) = 0.468  Compound: P = 0.097, F (1, 14) = 3.172  Time x Compound: P = 0.689, F (1, 14) = 0.167  baseline vs treatment  Vehicle: P = 0.905  Ulotaront: P > 0.999 |
| **2F, right** | Vehicle: 8 cells  Ulotaront: 8 cells | Unpaired t-test  (two-tailed) | P = 0.874  (t_14_ = 0.162) |
| **2G** | Vehicle: 9 cells  Ulotaront: 8 cells | Two-way RM ANOVA followed by Bonferroni’s multiple comparison tests (across time, within compound) | Subject: P = 0.0002, F (15, 15) = 7.370  Time: P = 0.760, F (1, 15) = 0.097  Compound: P = 0.285, F (1, 15) = 1.227  Time x Compound: P = 0.858, F (1, 15) = 0.033  baseline vs treatment  Vehicle: P > 0.999  Ulotaront: P > 0.999 |
| **2H, right** | Vehicle: 9 cells  Ulotaront: 8 cells | Unpaired t-test  (two-tailed) | P = 0.410  (t_15_ = 0.847) |
| **2I** | Vehicle: 9 cells  Ulotaront: 8 cells | Two-way RM ANOVA followed by Bonferroni’s multiple comparison tests (across time, within compound) | Subject: P < 0.0001, F (15, 15) = 36.09  Time: P = 0.840, F (1, 15) = 0.042  Compound: P = 0.531, F (1, 15) = 0.412  Time x Compound: P = 0.182, F (1, 15) = 1.957  baseline vs treatment  Vehicle: P = 0.521  Ulotaront: P = 0.850 |
| **2J, right** | Vehicle: 9 cells  Ulotaront: 8 cells | Unpaired t-test  (two-tailed) | P = 0.210  (t_15_ = 1.310) |
| **3E** | 24 cells | Paired t-test (two-tailed) | P = 0.074  (t_23_ = 1.874) |
| **3F**  **(mean)** | Vehicle: 20 cells  Ulotaront: 24 cells | Unpaired t-test with Welch’s correction  (two-tailed) | P = 0.176  (t_28.37_ = 1.388) |
| **3F**  **(variance)** | Vehicle: 20 cells  Ulotaront: 24 cells | F test | P < 0.0001  (F=9.983, DFn=23, Dfd=19) |
| **3I, left** | Vehicle: 8 cells  Ulotaront: 8 cells | Mann Whitney test  (two-tailed) | P = 0.0003  [Sum of ranks: 99 (vehicle), 37 (ulotaront)]  (Mann-Whitney U = 1) |
| **3I, right** | Vehicle: 12 cells  Ulotaront: 16 cells | Mann Whitney test  (two-tailed) | P = 0.0061  [Sum of ranks: 116 (vehicle), 290 (ulotaront)]  (Mann-Whitney U = 38) |
| **3K, left** | 14 cells | Paired t-test (two-tailed) | P = 0.749  (t_13_ = 0.327) |
| **3K, right**  **(mean)** | Vehicle: 12 cells  RO5166017: 14 cells | Unpaired t-test with Welch’s correction  (two-tailed) | P = 0.679  (t_14.26_ = 0.422) |
| **3K, left**  **(variance)** | Vehicle: 12 cells  RO5166017: 14 cells | F test | P < 0.0001  (F=23.88, DFn=13, Dfd=11) |
| **3M, top** | Vehicle: 6 cells  RO5166017: 7 cells | Mann Whitney test  (two-tailed) | P = 0.0023  [Sum of ranks: 62 (vehicle), 29 (RO5166017)]  (Mann-Whitney U = 1) |
| **3M, bottom** | Vehicle: 6 cells  RO5166017: 7 cells | Mann Whitney test  (two-tailed) | P = 0.014  [Sum of ranks: 25 (vehicle), 66 (RO5166017)]  (Mann-Whitney U = 4) |
| **4D** | Vehicle: 10 slices  10 μM ulotaront: 14 slices  30 μM ulotaront: 12 slices | Two-way RM ANOVA followed by Bonferroni’s multiple comparison tests (across time, within compound) | Subject: P < 0.0001, F (33, 33) = 28.34  Time: P = 0.0458, F (1, 33) = 3.822  Compound: P = 0.855, F (2, 33) = 0.157  Time x Compound: P = 0.666, F (2, 33) = 0.412  baseline vs treatment  Vehicle: P = 0.969  Ulotaront 10 μM: P = 0.190  Ulotaront 30 μM: P = 0.047 |
| **4E, right** | Vehicle: 10 slices  10 μM ulotaront: 14 slices  30 μM ulotaront: 12 slices | one-way ANOVA followed by Dunnett’s multiple comparison tests (against vehicle) | P = 0.028  F (2, 33) = 3.993  P = 0.565 (vehicle vs 10 μM ulotaront)  P = 0.019 (vehicle vs 30 μM ulotaront) |
| **4F** | Vehicle: 10 slices  10 μM ulotaront: 14 slices  30 μM ulotaront: 12 slices | Two-way RM ANOVA followed by Bonferroni’s multiple comparison tests (across time, within compound) | Subject: P < 0.0001, F (33, 33) = 80.85  Time: P = 0.0026, F (1, 33) = 10.62  Compound: P = 0.651, F (2, 33) = 0.435  Time x Compound: P = 0.048, F (2, 33) = 3.335  baseline vs treatment  Vehicle: P > 0.999  Ulotaront 10 μM: P = 0.541  Ulotaront 30 μM: P = 0.001 |
| **4G, right** | Vehicle: 10 slices  10 μM ulotaront: 14 slices  30 μM ulotaront: 12 slices | one-way ANOVA followed by Dunnett’s multiple comparison tests (against vehicle) | P = 0.032  F (2, 33) = 3.825  P = 0.307 (vehicle vs 10 μM ulotaront)  P = 0.018 (vehicle vs 30 μM ulotaront) |
| **5E** | Vehicle: 45 electrodes (8 slices)  1 μM ulotaront: 30 electrodes (6 slices)  10 μM ulotaront: 31 electrodes (8 slices)  30 μM ulotaront: 27 electrodes (7 slices) | Two-way RM ANOVA followed by Bonferroni’s multiple comparison tests (across time, within compound) | Subject: P < 0.0001, F (129, 129) = 56.94  Time: P < 0.0001, F (1, 129) = 18.49  Compound: P = 0.714, F (3, 129) = 0.455  Time x Compound: P = 0.0003, F (2, 129) = 6.647  baseline vs treatment  Vehicle: P > 0.999  Ulotaront 1 μM: P > 0.999  Ulotaront 10 μM: P = 0.017  Ulotaront 30 μM: P < 0.0001 |
| **5G** | Vehicle: 45 electrodes (8 slices)  1 μM ulotaront: 30 electrodes (6 slices)  10 μM ulotaront: 31 electrodes (8 slices)  30 μM ulotaront: 27 electrodes (7 slices) | one-way ANOVA followed by Dunnett’s multiple comparison tests (against vehicle) | P < 0.0001  F (3, 129) = 10.42  P = 0.9543 (vehicle vs 1 μM ulotaront)  P = 0.0059 (vehicle vs 10 μM ulotaront)  P < 0.0001 (vehicle vs 30 μM ulotaront) |
| **5K** | Vehicle: 9 cells  Ulotaront: 16 cells | Two-way RM ANOVA followed by Bonferroni’s multiple comparison tests (across time, within compound) | Subject: P < 0.0001, F (23, 23) = 20.31  Time: P = 0.300, F (1, 23) = 1.125  Compound: P = 0.685, F (1, 23) = 0.169  Time x Compound: P = 0.011, F (1, 23) = 7.687  baseline vs treatment  Vehicle: P = 0.591  Ulotaront: P = 0.008 |
| **5M** | Vehicle: 9 cells  30 μM ulotaront: 16 cells | Unpaired t-test  (two-tailed) | P = 0.0066  (t_23_ = 2.988) |
| **Supp 1C** | Baseline: 4075 events (9 cells)  Ulotaront: 2259 events (9 cells) | Kolmogorov-Smirnov test | P < 0.0001  K-S D = 0.2053 |
| **Supp 1D** | Baseline: 4878 events (8 cells)  Ulotaront: 2528 events (8 cells) | Kolmogorov-Smirnov test | P < 0.0001  K-S D = 0.2012 |
| **Supp 1E** | Baseline: 4075 events (9 cells)  Ulotaront: 2259 events (9 cells) | Kolmogorov-Smirnov test | P = 0.0005  K-S D = 0.055 |
| **Supp 1F** | Baseline: 4878 events (8 cells)  Ulotaront: 2528 events (8 cells) | Kolmogorov-Smirnov test | P < 0.0001  K-S D = 0.187 |
| **Supp 1G** | 8 cells | Paired t-test (two-tailed) | P = 0.214  (t_7_ = 1.368) |
| **Supp 1H, left (vehicle)** | 8 cells | Paired t-test (two-tailed) | P = 0.118  (t_7_ = 1.781) |
| **Supp 1H, right (ulotaront)** | 9 cells | Paired t-test (two-tailed) | P = 0.289  (t_8_ = 1.137) |
| **Supp 1I** | 8 cells | Paired t-test (two-tailed) | P = 0.320  (t_7_ = 1.070) |
| **Supp 1J, left (vehicle)** | 8 cells | Paired t-test (two-tailed) | P = 0.247  (t_7_ = 1.263) |
| **Supp 1J, right (ulotaront)** | 8 cells | Paired t-test (two-tailed) | P = 0.015  (t_7_ = 3.219) |
| **Supp 2C** | Baseline: 3329 events (8 cells)  Ulotaront: 3461 events (8 cells) | Kolmogorov-Smirnov test | P = 0.158  K-S D = 0.0274 |
| **Supp 2D** | Baseline: 2670 events (8 cells)  Ulotaront: 2635 events (8 cells) | Kolmogorov-Smirnov test | P = 0.051  K-S D = 0.0455 |
| **Supp 2E** | Baseline: 3329 events (8 cells)  Ulotaront: 3461 events (8 cells) | Kolmogorov-Smirnov test | P = 0.978  K-S D = 0.0122 |
| **Supp 2F** | Baseline: 2670 events (8 cells)  Ulotaront: 2635 events (8 cells) | Kolmogorov-Smirnov test | P = 0.363  K-S D = 0.0267 |
| **Supp 2G, left (CV^2^)** | 8 cells | Paired t-test (two-tailed) | P = 0.204  (t_7_ = 1.401) |
| **Supp 2G, right (VMR)** | 8 cells | Paired t-test (two-tailed) | P = 0.268  (t_7_ = 1.203) |
| **Supp 2H, left (CV^2^)** | 8 cells | Paired t-test (two-tailed) | P = 0.691  (t_7_ = 0.415) |
| **Supp 2H, right (VMR)** | 8 cells | Paired t-test (two-tailed) | P = 0.794  (t_7_ = 0.271) |
| **Supp 3C** | 10 cells | Paired t-test (two-tailed) | P = 0.265  (t_9_ = 1.190) |
| **Supp 3E**  **(mean)** | Vehicle: 11 cells  Ulotaront: 10 cells | Unpaired t-test with Welch’s correction  (two-tailed) | P = 0.238  (t_10.69_ = 1.251) |
| **Supp 3E**  **(variance)** | Vehicle: 11 cells  Ulotaront: 10 cells | F test | P = 0.0015  (F=9.654, DFn=9, Dfd=10) |
| **Supp 3F, top** | Vehicle: 7 cells  Ulotaront: 7 cells | Unpaired t-test  (two-tailed) | P = 0.105  (t_12_= 1.756) |
| **Supp 3F, bottom** | Vehicle: 4 cells  Ulotaront: 3 cells | Mann Whitney test  (two-tailed) | P = 0.114  [Sum of ranks: 21 (vehicle), 7 (ulotaront)]  (Mann-Whitney U = 1) |
| **Supp 4A, left** | 10 cells | Paired t-test (two-tailed) | P = 0.143  (t_9_ = 1.605) |
| **Supp 4A, right**  **(mean)** | Vehicle: 11 cells  Ulotaront: 10 cells | Unpaired t-test with Welch’s correction  (two-tailed) | P = 0.359  (t_12.55_ = 0.9514) |
| **Supp 4A, right**  **(variance)** | Vehicle: 11 cells  Ulotaront: 10 cells | F test | P = 0.058  (F=3.490, DFn=9, Dfd=10) |
| **Supp 4B, left** | 23 cells | Paired t-test (two-tailed) | P = 0.309  (t_22_ = 1.042) |
| **Supp 4B, right**  **(mean)** | Vehicle: 20 cells  Ulotaront: 23 cells | Unpaired t-test with Welch’s correction  (two-tailed) | P = 0.863  (t_40.60_ = 0.1743) |
| **Supp 4B, right**  **(variance)** | Vehicle: 20 cells  Ulotaront: 23 cells | F test | P = 0.853  (F=1.092, DFn=22, Dfd=19) |
| **Supp 4C, left** | 10 cells | Paired t-test (two-tailed) | P = 0.574  (t_9_ = 0.584) |
| **Supp 4C, right**  **(mean)** | Vehicle: 11 cells  Ulotaront: 10 cells | Unpaired t-test with Welch’s correction  (two-tailed) | P = 0.208  (t_16.84_ = 1.311) |
| **Supp 4C, right**  **(variance)** | Vehicle: 11 cells  Ulotaront: 10 cells | F test | P = 0.162  (F=2.628, DFn=10, Dfd=9) |
| **Supp 4D, left** | 24 cells | Paired t-test (two-tailed) | P = 0.858  (t_23_ = 0.1805) |
| **Supp 4D, right**  **(mean)** | Vehicle: 20 cells  Ulotaront: 24 cells | Unpaired t-test with Welch’s correction  (two-tailed) | P = 0.081  (t_32.92_ = 1.802) |
| **Supp 4D, right**  **(variance)** | Vehicle: 20 cells  Ulotaront: 24 cells | F test | P = 0.096  (F=2.078, DFn=19, Dfd=23) |
| **Supp 4G, left** | 10 cells | Paired t-test (two-tailed) | P = 0.0582  (t_9_ = 2.084) |
| **Supp 4G, right**  **(mean)** | Vehicle: 11 cells  Ulotaront: 10 cells | Unpaired t-test with Welch’s correction  (two-tailed) | P = 0.210  (t_18.97_ = 1.297) |
| **Supp 4G, right**  **(variance)** | Vehicle: 11 cells  Ulotaront: 10 cells | F test | P = 0.862  (F=1.131, DFn=10, Dfd=9) |
| **Supp 4H, left** | 24 cells | Paired t-test (two-tailed) | P = 0.226  (t_23_ = 1.244) |
| **Supp 4H, right**  **(mean)** | Vehicle: 20 cells  Ulotaront: 24 cells | Unpaired t-test with Welch’s correction  (two-tailed) | P = 0.555  (t_35.27_ = 0.5961) |
| **Supp 4H, right**  **(variance)** | Vehicle: 20 cells  Ulotaront: 24 cells | F test | P = 0.0038  (F=3.906, DFn=23, Dfd=19) |
| **Supp 5B, left** | 14 slices | Paired t-test (two-tailed) | P = 0.087  (t_13_ = 1.852) |
| **Supp 5B, right** | 12 slices | Paired t-test (two-tailed) | P = 0.171  (t_11_ = 1.465) |
| **Supp 5C** | Vehicle: 10 slices  10 μM ulotaront: 14 slices  30 μM ulotaront: 12 slices | one-way ANOVA | P = 0.376  F (2, 33) = 1.008 |
| **Supp 5D, left** | 14 slices | Paired t-test (two-tailed) | P = 0.223  (t_13_ = 1.281) |
| **Supp 5D, right** | 12 slices | Paired t-test (two-tailed) | P = 0.291  (t_11_ = 1.110) |
| **Supp 5E** | Vehicle: 10 slices  10 μM ulotaront: 14 slices  30 μM ulotaront: 12 slices | one-way ANOVA | P = 0.814  F (2, 33) = 0.2073 |
